# Intrabody against prolyl hydroxylase 2 promotes angiogenesis by stabilizing hypoxia-inducible factor-1α

**DOI:** 10.1038/s41598-019-47891-1

**Published:** 2019-08-14

**Authors:** Liangzhong Zhao, Ziyu Liu, Fang Yang, Ying Zhang, Ying Xue, Haipeng Miao, Xiangzhi Liao, Hongli Huang, Guiying Li

**Affiliations:** 1 Key Laboratory for Molecular Enzymology and Engineering of the Ministry of Education, School of Life Sciences, Jilin University, Changchun, 130012 China; 2 Department of Immunoassay Technology, Jilin Medical University, Jilin, 132013 China; 3 Department of Pediatrics, The First Hospital of Jilin University, Changchun, Jilin 130021 P.R. China

**Keywords:** Cell biology, Molecular medicine

## Abstract

Hypoxia-inducible factor (HIF)-1α is a crucial transcription factor that regulates the expression of target genes involved in angiogenesis. Prolyl hydroxylase 2 (PHD2) dominantly hydroxylates two highly conserved proline residues of HIF-1α to promote its degradation. This study was designed to construct an intrabody against PHD2 that can inhibit PHD2 activity and promote angiogenesis. Single-chain variable fragment (scFv) against PHD2, INP, was isolated by phage display technique and was modified with an endoplasmic reticulum (ER) sequence to obtain ER-retained intrabody against PHD2 (ER-INP). ER-INP was efficiently expressed and bound to PHD2 in cells, significantly increased the levels of HIF-1α, and decreased hydroxylated HIF-1α in human embryonic kidney cell line (HEK293) cells and mouse mononuclear macrophage leukaemia cell line (RAW264.7) cells. ER-INP has shown distinct angiogenic activity both *in vitro* and *in vivo*, as ER-INP expression significantly promoted the migration and tube formation of human umbilical vein endothelial cells (HUVECs) and enhanced angiogenesis of chick chorioallantoic membranes (CAMs). Furthermore, ER-INP promoted distinct expression and secretion of a range of angiogenic factors. To the best of our knowledge, this is the first study to report an ER-INP intrabody enhancing angiogenesis by blocking PHD2 activity to increase HIF-1α abundance and activity. These results indicate that ER-INP may play a role in the clinical treatment of tissue injury and ischemic diseases in the future.

## Introduction

There are three members of the hypoxia-inducible factor (HIF) family: HIF-1, HIF-2, and HIF-3. HIFs are heterodimers that share the same constitutively expressed β subunit (HIF-1β or ARNT), but have different oxygen-sensitive α subunits (HIF-1α, HIF-2α, and HIF-3α, respectively)^1–3^. HIF-1 is a crucial transcription factor that regulates the expression of target genes involved in angiogenesis and tissue repair after acute injury^4–6^. Under normoxic conditions, HIF-1α is rapidly hydroxylated on proline 402 (Pro402) and Pro564 by specific oxygen-dependent prolyl hydroxylases (PHDs) and then degraded by proteasomes via conjugation with the von Hippel–Lindau (VHL) E3 ubiquitin ligase complex^7,8^. PHDs (PHD1, PHD2, and PHD3) belong to the family of the dioxygenase enzymes that require oxygen, iron, and 2-oxyglutarate (2-OG) for their catalytic activity. Under hypoxic conditions, the activity of PHDs is reduced in a graded manner, but significant PHD activity is still observed at 1% oxygen because of their high affinity for oxygen^9,10^. Indeed, several reports have documented that HIFs still become hydroxylated under nearly completely anoxic conditions^11,12^.

In summary, manipulation of PHD levels or activity can be a key determinant of the hydroxylation rate of HIFα and directly affects the stability of HIFα^13^. More evidence has demonstrated that the inhibition of PHD2 activity increased the accumulation of HIF-1α, which then dimerised with HIF-1β to transactivate target genes^14,15^. HIF-1α controls various target genes involving angiogenesis, metabolic adaptation, and inflammation through directly binding to the promoter region of the targets^16^. Several reports demonstrated that the HIF-1-regulated gene vascular endothelial growth factor (*VEGF*) was identified as an important mediator of tissue repair during APAP hepatotoxicity^17,18^, liver graft injury^19^, and liver ischemic injury^20^. Thus, many small-molecule drug candidates have been proposed as PHD2 inhibitors with a wide range of potencies spanning from the discovery stage to clinical trials^21–24^. However, the PHD inhibitors currently used in clinical and research settings produce a large number of toxic side effects^25–27^. Therefore, it is imperative to obtain inhibitors that specifically inhibit PHD2 activity.

Antibodies have been widely used to inactivate target proteins because of their high specificity and affinity to target antigens^28^. Antibody-based therapy for cancer has become established over the past 20 years and is now one of the most successful and important strategies for treating patients with haematological malignancies and solid tumours^29^. Intrabodies are recombinant antibody fragments that bind to target proteins expressed inside of the same living cell^30^. Different types of intrabodies must be designed to target proteins at different locations, typically either in the cytoplasm, nucleus, or the endoplasmic reticulum (ER). Although intrabodies are typically seen as an experimental tool to reveal the function of proteins by interfering with their function, this approach has also been reported to have therapeutic potential against viral infections^31^, brain diseases^32^, and cancer^33^. In addition, anti-WASP intrabodies inhibit inflammatory responses induced by Toll-like receptors 3, 7, and 9 in macrophages; therefore, designing drugs that mimic the single-chain variable fragment (scFv) intrabody may result in new anti-inflammatory agents with fewer side effects^34^. The present study was designed to construct an ER-retained intrabody against PHD2 (ER-INP), which inhibited PHD2 activity and increased the stability of HIF-1α. Accordingly, we explored the effects of ER-INP on the downstream molecules of HIF-1α and investigated the role of ER-INP in angiogenesis mediated by HIF-1α. The results suggest that ER-INP may provide a new potential strategy for the treatment of tissue injury and angiogenesis-related diseases.

## Results

### Expression and distribution of ER-INP in cells

**Figure 1:**
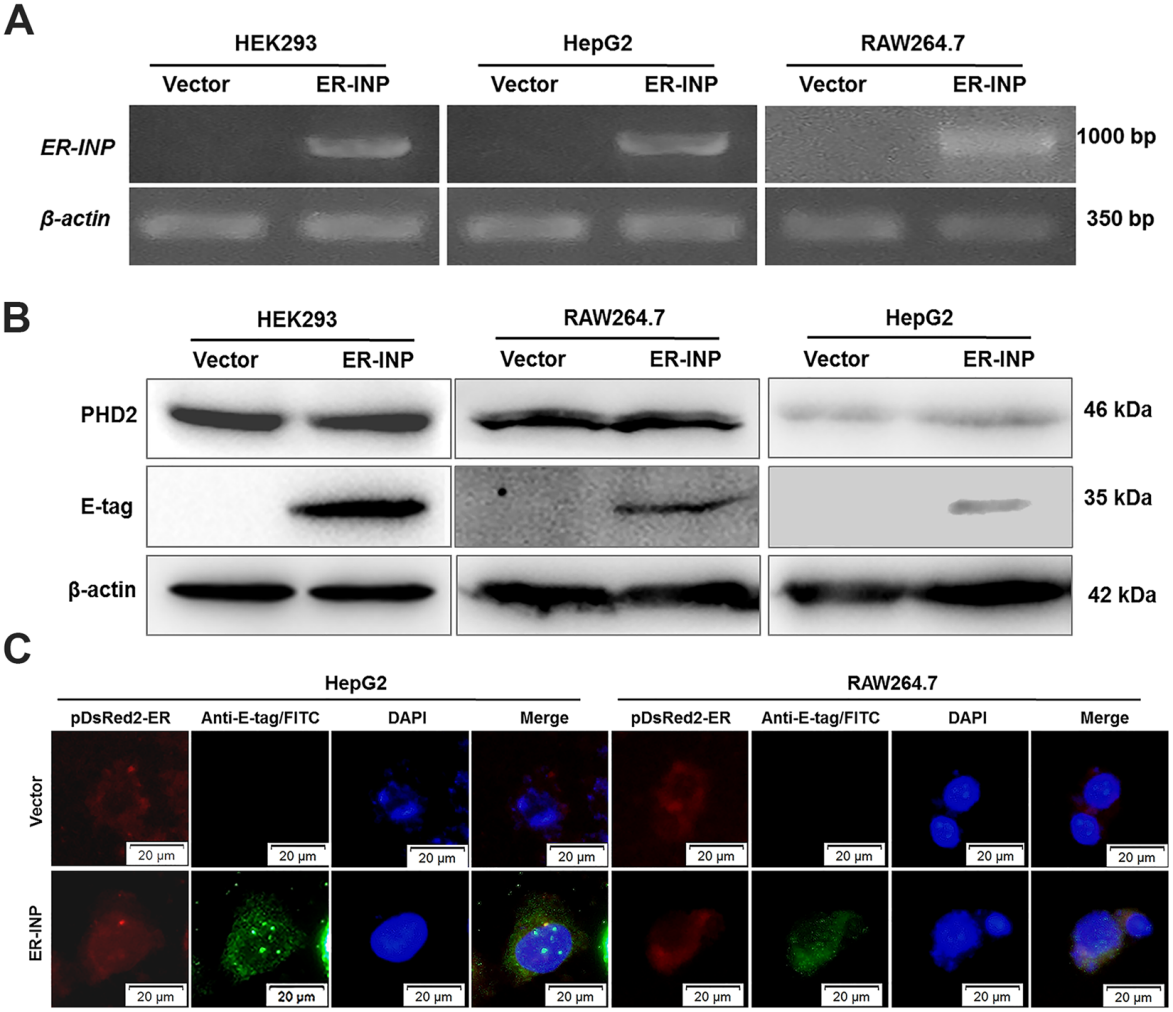
Expression of ER-INP in cells. (**A**) Expression of ER-INP in cells detected by RT-PCR (n = 3). (**B**) Protein expression of ER-INP in cells and the effect of ER-INP expression on the level of PHD2 protein in cells, as determined by western blot (n = 3). **(C)** Intracellular localization of ER-INP by immunofluorescence assay. Cells were co-transfected with pDsRed2-ER and pER-INP for 48 hours and stained with an anti-E-tag antibody to visualize the intrabody ER-INP and pDsRed2-ER as an ER marker, then observed by immunofluorescence microscopy. DsRed2-ER and ER-INP co-localized in the endoplasmic reticulum (n = 3).

PHD2 is regarded as the main cellular oxygen sensor, as inhibition of PHD2 activity is enough to stabilize HIF-1α in normoxia^35^. PHD2 was expected to be present in the cytoplasm and nucleus, but the majority of it was actually found in the cytoplasm^36^. The immunofluorescence (IF) results demonstrated that PHD2 was also mainly localized in the cytoplasm of HepG2 human hepatoma cells (Supplementary Fig. 1). To verify the expression of the constructed ER-INP intrabody, HEK293 (human embryonic kidney cell line), HepG2, and RAW264.7 (mouse mononuclear macrophage leukaemia cell line) cells were transfected with either the expression plasmid of ER-INP (pER-INP) or the control vector plasmid. RT-PCR results demonstrated that ER-INP was expressed successfully in cells transfected with pER-INP compared with that in the control vector (Fig. 1A). Western blots of cell lysates with the E-tag antibody showed that ER-INP fused with the E-tag was expressed with an estimated molecular weight of about 35 kDa in HEK293, HepG2, and RAW264.7 cells; meanwhile, the expression of ER-INP had no effect on the expression of PHD2 in transfected cells (Fig. 1B). We further investigated the intracellular distribution of ER-INP in HepG2 and RAW264.7 cells by IF analysis. Cells were cotransfected with pER-INP or the control vector and pDsRed2-ER expressing an ER marker. ER-INP was detected by anti-E-tag antibody and FITC (green fluorescence), and the expression and localization of ER-INP and DsRed2-ER were analysed by fluorescence microscopy. We observed that control cells only had red fluorescence and no green fluorescence signals. This indicated that there was no ER-INP expression and no colocalization with DsRed2-ER in the control cells. In contrast, ER-INP was expressed and co-localized with DsRed2-ER in the ER of the cells cotransfected with pER-INP and pDsRed2-ER, as both bright red fluorescence and green fluorescence signals in these cells were observed (Fig. 1C). However, we did not obtain IF data for HEK293 cells since the HEK293 cells easily washed away from the slides during experimental operation. In addition, ER-INP was co-localized with PHD2 in the ER of the cells, as we also observed the overlap of bright red fluorescence and green fluorescence signals in HepG2 cells (Supplementary Fig. 2). These results show that ER-INP was efficiently expressed and localized in the ER, which is consistent with our experimental design.

### ER-INP binds to PHD2 and inhibits PHD2 activity

**Figure 2:**
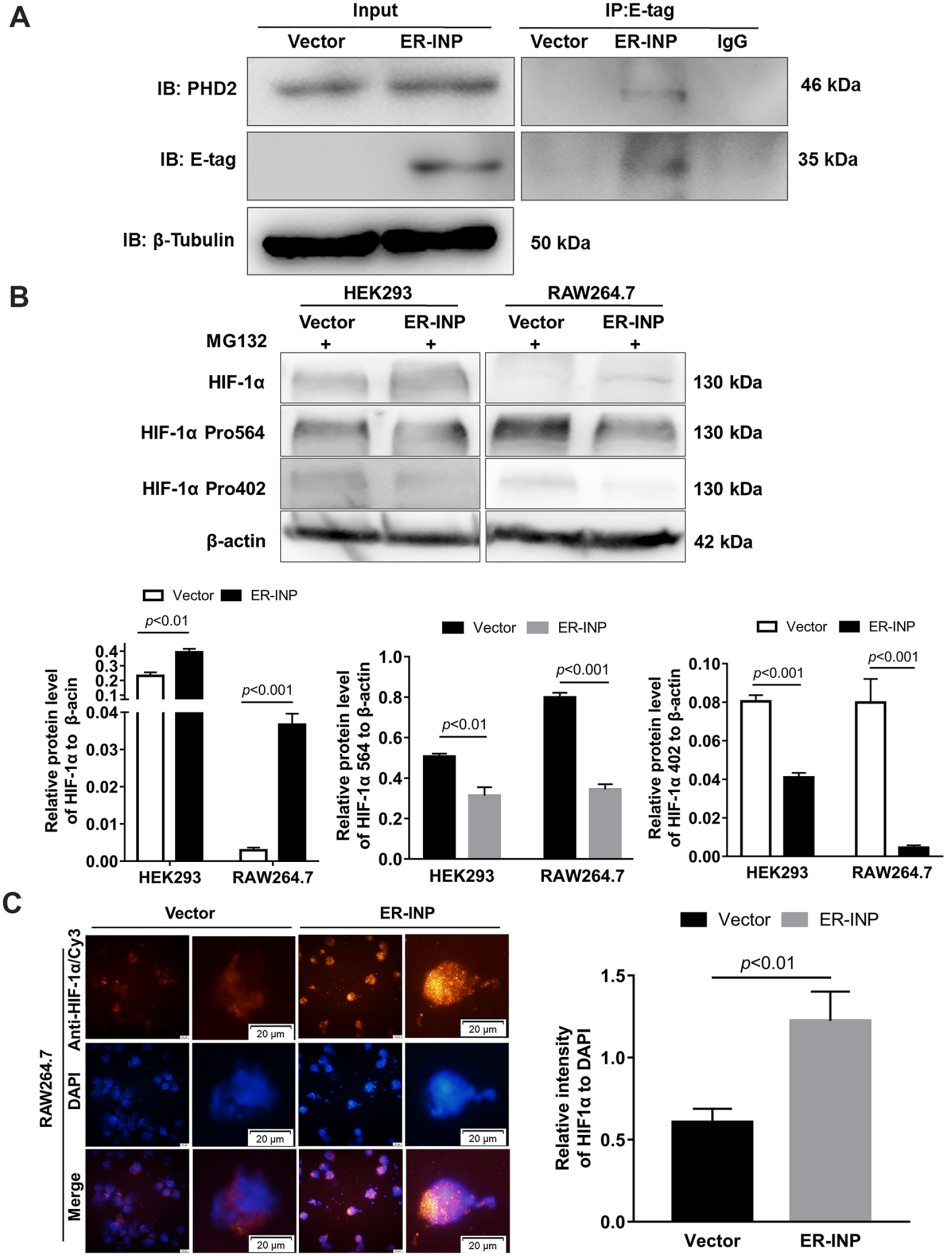
ER-INP binds to PHD2, inhibits hydroxylation of HIF-1α and increases HIF-1α accumulation. (**A)** Co-immunoprecipitation assay. HEK293 cells were transfected with control vector or intrabody ER-INP for 48 hours and subsequently lysed. Co-immunoprecipitation and western blot assays were performed on the cell lysis. ER-INP recognized and bound to PHD2 in HEK293 cells (n = 3). **(B)** Western blot analysis to measure the effect of ER-INP on HIF-1α and its hydroxylation level in transfected HEK293 and RAW264.7 cells pre-treated with MG132 (upper panel) and the protein ratio to β-actin loading control by ImageJ densitometry analysis (lower panel; n = 3). **(C)** Immunofluorescence assay. Expression of ER-INP increases HIF-1α accumulation in RAW264.7 cells. Cells were stained with anti-HIF-1α antibody and DAPI and then visualized and photographed under immunofluorescence microscopy (left), and mean fluorescence intensity of HIF-1α versus the mean fluorescence intensity of nuclear DAPI staining is displayed (right). Data represent the mean ± SD of 3 independent slides.

To determine whether ER-INP binds to PHD2 intracellularly, the interaction between PHD2 and its specific ER-INP intrabody was further investigated by a co-immunoprecipitation assay. Cells were transiently transfected with pER-INP or the control vector. At 48 hours post-transfection, cell lysates were prepared and immunoprecipitated with anti-E-tag monoclonal antibodies and analysed by western blot with anti-PHD2 polyclonal antibodies and anti-E-tag monoclonal antibodies. The results demonstrated that ER-INP intrabody molecules could recognize and bind to PHD2 in HEK293 cells (Fig. 2A). To gain insight into the regulation of HIF-1α abundance, we further determined HIF-1α protein levels in HEK293 and RAW264.7 cells transfected with pER-INP or the control vector under normoxia. To prevent the degradation of HIF-1α, transfected cells were pre-treated with MG132, a proteasome inhibitor known to retard proteasome-dependent protein degradation^37^. We discovered that ER-INP notably elevated HIF-1α protein levels in HEK293 and RAW264.7 cells. In contrast, no appreciable induction of HIF-1α was observed in cells treated with the control vector (Fig. 2B). To explore how ER-INP elevated HIF-1α levels, we firstly determined the mRNA levels of HIF-1α and found that there was no change at all following ER-INP treatment in HEK293 and RAW264.7 cells (data not shown). Given that PHD2 is the rate-limiting enzyme mediating the degradation of HIF-1α by hydroxylation of HIF-1α at Pro564 and Pro402^7,8^, we thus hypothesized that ER-INP impaired the degradation of HIF-1α. We used specific antibodies that recognized HIF-1α hydroxylated at Pro564 and Pro402 to determine the levels of hydroxylated HIF-1α when proteasomal degradation was inhibited by MG132. Compared with the control groups, expression of ER-INP dramatically reduced the levels of hydroxylated HIF-1α (Fig. 2B). Notably, the total HIF-1α abundance was inversely correlated with the levels of hydroxylated HIF-1α. Furthermore, we also assessed the levels of HIF-1α protein by IF assay. As anticipated, ER-INP expression significantly enhanced HIF-1α fluorescence intensity versus nuclear staining when compared with that observed in control cells (Fig. 2C; *P* < 0.01). These results indicate that ER-INP binds specifically to PHD2 and blocks the hydroxylation of HIF-1α at Pro564 and Pro402, thereby stabilizing HIF-1α. This observation was consistent with the previous reports that HIF-1α stability was primarily regulated by PHD2^14,38^.

### ER-INP promotes migration and tube formation of HUVECs *in vitro*

**Figure 3:**
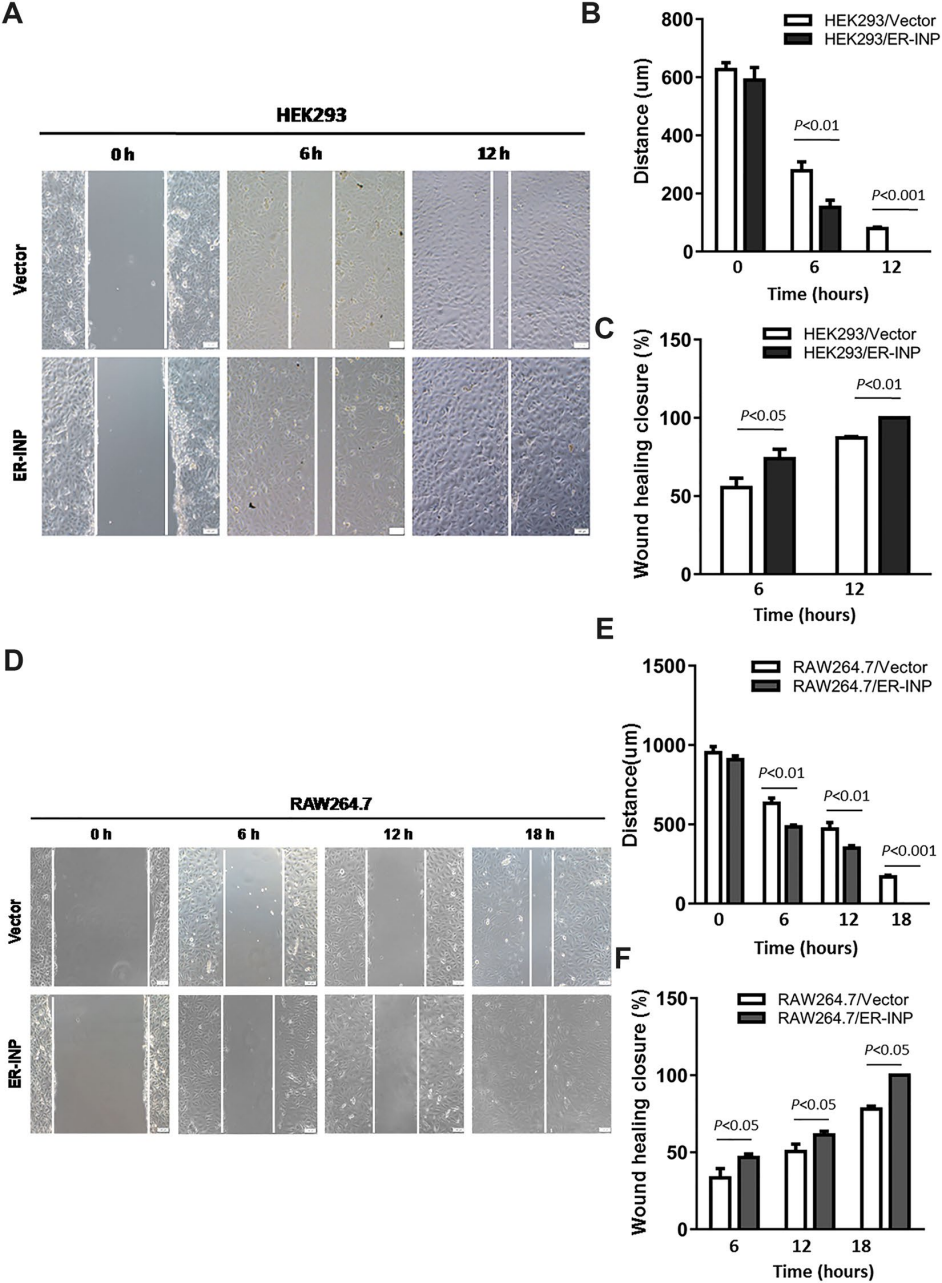
ER-INP increases migratory capacity of HUVECs. HUVECs were co-cultured with transfected HEK293 cells **(A–C)** and RAW26.4 cells **(D–F)** for different lengths of time. Then, the migratory capacity of HUVECs was observed under a microscope **(A**,**D)** and analysed **(B**,**C**,**E**,**F)**. The quantitative charts of scratch width **(B**,**E)**. The quantitative charts of scratch closure rate **(C**,**F)**. ER-INP increases the migratory capacity of HUVECs. Data represent the mean ± SD from 3 parallel experiments of representative data from multiple independent experiments **(B**,**C**,**E**,**F)**.

HIF-1α, which is the primary physiologic regulator of VEGF-mediated angiogenesis, directs further production of excess VEGF in response to stimulatory HIF-1α stabilization pathways^39^. It is also well established that the migration and tube formation of vascular endothelial cells play critical roles in angiogenesis^40^. To investigate whether ER-INP regulates angiogenesis, we first expressed ER-INP in HEK293 and RAW264.7 cells and examined the effect of ER-INP on the biological characteristics of human umbilical vein endothelial cells (HUVECs). The migration of HUVECs was examined by the wound healing assay. Compared to the control vector group, HUVECs co-cultured with HEK293 or RAW264.7 cells transfected with pER-INP displayed obviously increased migratory capacity at different time points (Fig. 3). Moreover, the width of the HUVEC scratches decreased (*P* < 0.01) while the scratch closure rate increased (*P* < 0.05). Additionally, culture medium alone (blank control) was added to the inserts and co-cultured with HUVECs and did not display any migratory capacity (data not shown). Thus, ER-INP promotes the migration of HUVECs.

**Figure 4:**
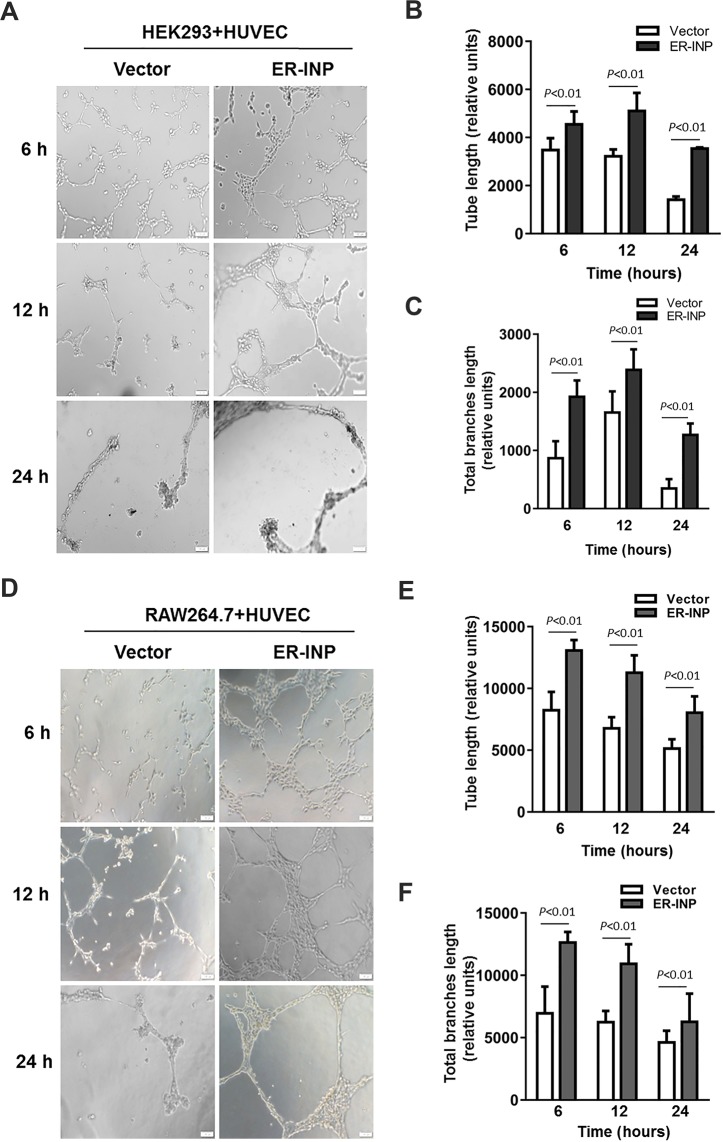
Tube formation assay of HUVECs. HUVECs were co-cultured with transfected HEK293 cells **(A–C)** and RAW26.4 cells **(D–F)** for different lengths of time. Then, the capacity of tube formation of HUVECs was observed under a microscope **(A**,**D)** and analysed by ImageJ software **(B**,**C**,**E**,**F)**. Tube length **(B**,**E)**. Total branch length **(C**,**F)**. ER-INP increases the capacity of tube formation of HUVECs. Data represent the mean ± SD from 3 parallel experiments of representative data from multiple independent experiments **(B**,**C**,**E**,**F)**.

Tube formation of HUVECs was determined by the three-dimensional capillary tube formation assay, which has been used as a model of the early organisation of new blood vessels and has consistently been found to replicate the process of angiogenesis *in vivo* to a great extent^41,42^. When co-cultured with HEK293 or RAW264.7 cells transfected with pER-INP, HUVECs showed improved capacity of tube formation in Matrigel (Fig. 4), and tube length and total branch length of tubes clearly increased when compared to the control vector group (*P* < 0.01). Obviously, tube formation processes were enhanced at 6 hours, but these processes ceased at 24 hours and the formed tubes disappeared in the control vector group (Fig. 4), consistent with previous papers^43^. However, upon co-culturing with culture medium alone (blank control), HUVECs exhibited no significant tube formation (data not shown). The data from these *in vitro* studies showed that ER-INP largely enhanced HUVECs migration and tube formation, which are essential for angiogenesis.

### ER-INP promotes angiogenesis of CAM

**Figure 5:**
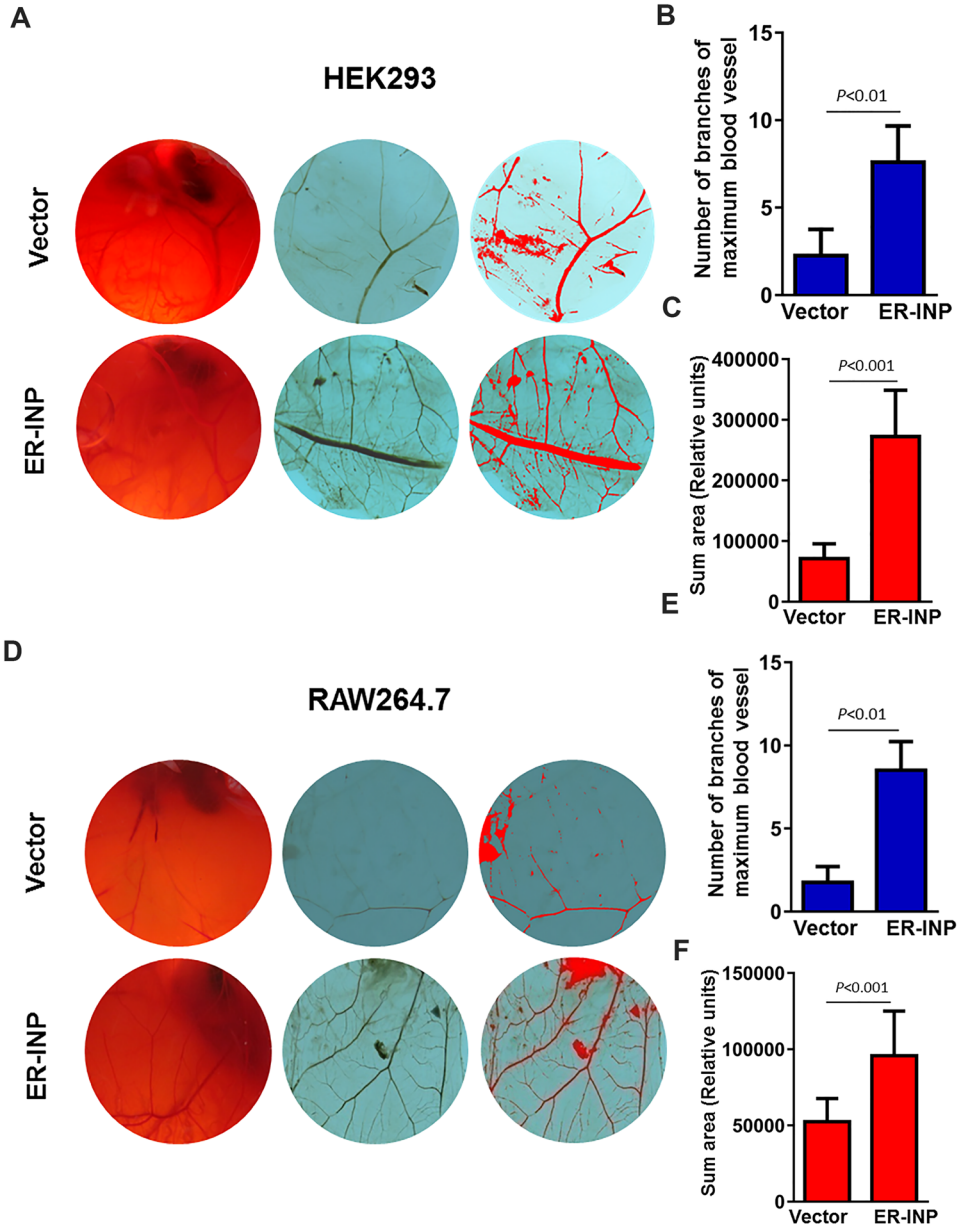
Expression of ER-INP promotes angiogenesis of chick chorioallantoic membranes (CAMs). Culture supernatant of cells transfected with ER-INP or the control vector was subjected to a CAM assay to determine the effect of ER-INP on angiogenesis. CAM blood vessels were observed in the entire field and photographed **(A**,**D)**. Representative images of CAMs. Number of branches of maximum blood vessel were counted **(B**,**E)** and the summed area was measured by Image Pro Plus statistics software **(C**,**F)**. Data represent the mean ± SD from 6 eggs for each group **(B**,**C**,**E**,**F)**.

To determine whether ER-INP exerts proangiogenic activity and to further our understanding of how ER-INP promotes the angiogenesis process, we carried out the chick chorioallantoic membrane (CAM) assay. CAM is a highly vascularized membrane and its position is directly under the inner surface of the egg shell. CAM has been used to support xenograft development because CAM can supply blood vessels to the xenograph^44^. The CAM is a widely used model for studying angiogenesis^45^. We exposed the CAM surface to the culture supernatant of HEK293 and RAW264.7 cells transfected with pER-INP and the control vector, and incubated them for 48 hours. When compared with the eggs treated with the culture supernatant of cells transfected with the control vector, eggs treated with ER-INP strongly elicited an angiogenic response, forming lots of new capillaries from the existing vascular network. ER-INP not only increased the number of branches of the maximum blood vessel (*P* < 0.01), but also expanded the summed area of blood vessels of CAM (*P* < 0.01; Fig. 5). Additionally, the eggs treated with culture medium alone (blank control) showed clear vascular texture and orderly structure (data not shown). These results support that ER-INP acts as an inducer of angiogenesis *in vivo*.

### ER-INP upregulates the expression and secretion of angiogenesis-related factors of cells

**Figure 6:**
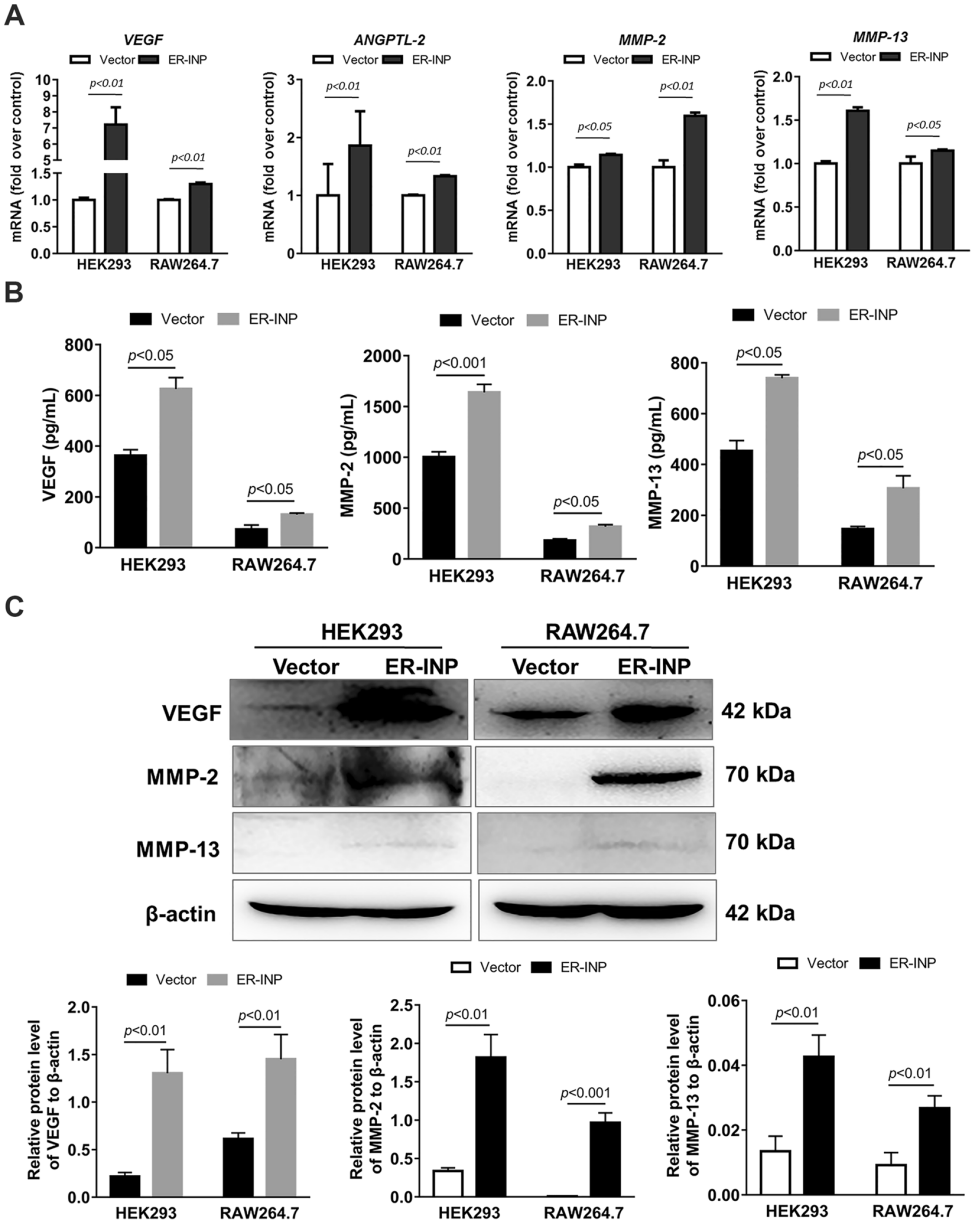
ER-INP upregulates the expression and secretion of angiogenesis-related factors of cells. **(A)** RT-qPCR analysis to measure the mRNA expression levels of angiogenesis-related genes in transfected HEK293 and RAW264.7 cells. **(B)** ELISA assay to determine the concentration of VEGF, MMP-2, and MMP-13 in cell culture supernatants from transfected HEK293 and RAW264.7 cells. **(C)** Western blot analysis to measure VEGF, MMP-2, and MMP-13 protein levels in transfected HEK293 and RAW264.7 cells (upper panel) and the protein ratio to β-actin loading control by ImageJ densitometry analysis (lower panel) (n = 3). Data represent the mean ± SD of 3 independent experiments.

To further explore the potential mechanisms by which ER-INP regulates the angiogenesis process, we analyzed the mRNA, protein expression, and cell culture supernatant levels of angiogenesis-related factors in HEK293 and RAW264.7 cells by RT-qPCR, western blot, and ELISA analyses, respectively. Compared with the control vector group, HEK293 and RAW264.7 cells transfected with pER-INP showed higher mRNA levels of a number of angiogenic genes including *VEGF*, angiopoietin-like protein 2 (*ANGPTL-2*), and matrix metallopeptidase 2 (*MMP-2*), which are identified downstream genes of HIF-1α (Fig. 6A). Subsequent western blot analysis in our study revealed that protein levels of VEGF and MMP-2 were significantly higher in the HEK293 and RAW264.7 cells transfected with pER-INP than that in the corresponding control vectors (Fig. 6C). We also found that MMP-13 mRNA and protein levels were upregulated in HEK293 and RAW264.7 cells transfected with pER-INP compared with those of the corresponding control vectors (Fig. 6A,C). Ha *et al*. also suggested that MMP-13 has an angiogenic effect and that MMP-13 is regulated by HIF-1α^46^.

We further investigated whether ER-INP could promote migration, tube formation of HUVECs, and exhibit high angiogenic activity of CAM possibly due to some soluble mediator released from the HEK293 or RAW264.7 cells where PHD2 activity is blocked. At 48 hours post-transfection, cell culture supernatant was analyzed by an ELISA assay according to the manufacturer’s instructions. Secretion of VEGF, MMP-2, and MMP-13 were significantly elevated by ER-INP in HEK293 and RAW264.7 cells when compared to the corresponding control vector (Fig. 6B). These results suggest that the effect ER-INP has on increasing angiogenesis must be due to some pro-angiogenic mediators regulated by HIF-1α, and these pro-angiogenic mediators are involved in driving migration and tube formation in the HUVECs.

Taken together, the above results show that ER-INP recognizes and binds to PHD2 and then blocks the hydroxylase activity of PHD2. Thus, ER-INP induces HIF-1α accumulation and the activation of downstream angiogenesis-related genes, resulting in significant pro-angiogenesis effects.

## Discussion

In this present study, we developed a novel ER-retained intrabody, ER-INP. ER-INP was identified by its ability to bind and block PHD2 activity. ER-INP expression significantly upregulated HIF-1α protein levels in HEK293 and RAW264.7 cells by inhibiting hydroxylation of HIF-1α. ER-INP promotes angiogenic responses of endothelial cells by activating HIF-1α and its target genes, such as *VEGF*, *ANGPTL-2*, *MMP-2*, and *MMP-*13. To our knowledge, this is the first ER-retained scFv intrabody that robustly enhances angiogenesis by blocking intracellular PHD2 activity and increasing HIF-1α level and activity. The present study provides a new strategy for targeting intracellular PHD2. The intrabody against PHD2 may play an important role in clinical improvement of angiogenesis.

Increasing evidence has shown that angiogenesis is an integral part of tissue repair and regeneration because functional microvessels facilitate the delivery of nutrition, oxygen, and infiltrating immune cells to the damaged tissue^5^. HIF-1α is a transcription factor that plays a crucial role in regulating the expression of target genes involved in angiogenesis of the damaged tissue^47^. The stability of the HIF-1α protein is tightly controlled by O_2_ availability. PHD2 is known to serve as the critical oxygen sensor, setting the low steady-state levels of HIF-1α^14^. Previous reports demonstrated that mRNA and protein levels of PHD2 increased after hypoxia^48,49^, which indicates that blocking PHD2 activity is useful to stabilize HIF-1α under either normoxic or hypoxic conditions. Thereby, PHD2 is emerging as an attractive drug target to be blocked for the treatment of hypoxic and ischemic diseases, including acute tissue injury, organ failure, acute anemia, and inflammation^35,50,51^. Loinard *et al*. demonstrated that a direct inhibition of PHDs promoted therapeutic revascularization, and subsequent activation of the HIF-1 signaling pathway is required to promote this revascularization^50^. Koivunen *et al*. suggested that PHD inhibition showed a therapeutic role in anemia, cardiac ischemia, obesity and metabolic dysfunction, and atherosclerosis by stabilizing HIF to promote glucose intake and glycolysis over oxidative metabolism^35^. Moreover, Tomohiro *et al*. showed that inhibition of the oxygen sensor PHD2 in the liver improves survival in lactic acidosis by activating the Cori cycle in an endotoxin shock mice model^51^. All the literature of PHD regulation of HIF-1α-mediated protection suggested that PHDs are attractive therapeutic targets. The regulation of HIF-1α is a pivotal element for anemia, ischemic diseases, tissue injuries, and inflammatory diseases.

Genetically engineered antibodies refer to recombinant antibody molecules which contain at least one complete or partial antigen-binding site to retain antigen-binding function. scFv is a key small molecule of engineered antibodies with a small size of ∼25 kDa, high affinity, and low immunogenicity^28^. scFv-based intrabody technology has emerged as a novel tool to modulate the function of intracellular proteins^52^. In contrast to the naturally expressed antibodies which are secreted and directed towards extracellular targets, intracellularly expressed antibodies are directed towards targets inside the cell. High specificity of antibody–antigen binding allows this to be utilized for the functional analysis of proteins as well as many therapeutic applications^53^. Knockdown of cellular proteins by intrabodies has been reported for a significant number of disease-relevant targets, including ErbB-2, EGFR, VEGFR2, MMP-2, and many others^52^. Since PHD2 mainly primes HIF-1α for degradation, we have made an effort to construct an ER-INP intrabody against PHD2 to explore whether it is capable of upregulating the HIF pathway by inhibiting prolyl hydroxylation. The data has shown that ER-INP activates the HIF-1α pathway and boosts its ability for angiogenesis by blocking PHD2 activity. ER-INP may be a novel approach for the treatment of hypoxic and ischemic diseases.

Because the folding of naturally-secreted antibodies is optimal in the ER^52^, we constructed an ER-retained intrabody against PHD2 (ER-INP), utilizing the amino acid sequence SEKDEL, which includes a C-terminal ER retention signal attached to the specific scFv antibody^54^. ER-retained intrabodies result in knockdown of their target proteins passing through the ER, blocking activation of downstream pathways^30^. ER-INP can bind to PHD2 and implements the functional knockdown of PHD2 that passes through the ER. This in turn induces the accumulation of HIF-1α and its translocation to the nucleus, initiating the signaling pathways that ultimately lead to angiogenesis.

Therefore, we examined the migration and tube formation of HUVECs in the co-cultures of HUVECs and HEK293 or RAW264.7 cells transfected with the control vector or pER-INP. The data from these *in vitro* studies showed that the ER-INP largely enhanced migration and tube formation of HUVECs. Furthermore, *in vivo* we found that the cell culture supernatants of HEK293/ER-INP and RAW264.7/ER-INP cells enhanced the angiogenesis of CAM compared with that in the corresponding controls. Based on these findings, we speculated that the improved angiogenesis effects must be due to some soluble mediator released from HEK293 or RAW264.7 cells where PHD2 activity is blocked. Qiang You *et al*. found that HIF may be involved in regulating the angiogenic effect of hepatic macrophages, which expressed dramatically higher levels of a number of angiogenic genes, including *VEGF*; *MMP-2*, *-7*, *-8*, *-9*, and -*13*; *ANGPTL2*; and *ANGTL4*, contributing to tissue recovery from acute injury^6^. According to this paper, we chose *VEGF*, *ANGPTL-2*, *MMP-2*, and *MMP-13* (which are key factors during angiogenesis) to confirm our hypothesis. *VEGF* is a potent angiogenic factor and an important target gene of HIF1 that mediates the survival, proliferation, migration, invasion, and permeability of endothelial cells (ECs)^47^. *MMP-2*, which is linked to HIF-1α expression, is considered to be critical in assisting cell migration and invasion during angiogenesis and metastasis^6^. *MMP-13* plays an important role in the matrix degradation process, which has been confirmed to promote tumour invasion, migration, and angiogenesis in a variety of tumours^55,56^. ANGPTL2, one of the proteins in the ANGPTL family that shares common domain characteristics with angiopoietins (ANGPTs), maintains tissue homeostasis by inducing inflammation and angiogenesis^6^.

Data presented in this study clearly demonstrated the expression of *VEGF*, *ANGPTL-2*, *MMP-2*, and *MMP-13* was elevated in ER-INP cells by RT-qPCR and the expression of VEGF, MMP-2, and MMP-13 was elevated in ER-INP cells by western blot assay. Compared with the control vector, ER-INP dramatically increased the concentration of secreted VEGF, MMP-2, and MMP-13 in cell culture supernatants, as observed in the ELISA assay. These results demonstrated that the mechanism of ER-INP involved in angiogenesis is attributed to the release of soluble mediators coded by downstream genes of HIF-1α in transfected HEK293 or RAW264.7 cells. Although MMP-13 is not transcriptionally activated by HIF-1α, previous studies have shown MMP-13 could be induced by VEGF, the latter known to be induced by HIF-1α^46,57^. Given that MMP-13 has been shown to play critical roles in endothelium-dependent angiogenesis, we speculated that MMP-13 combined with VEGF, ANGPTL-2, and MMP-2 to promote angiogenesis. However, elucidation of the exact pathway by which ER-INP controls angiogenesis needs further investigation.

**Figure 7:**
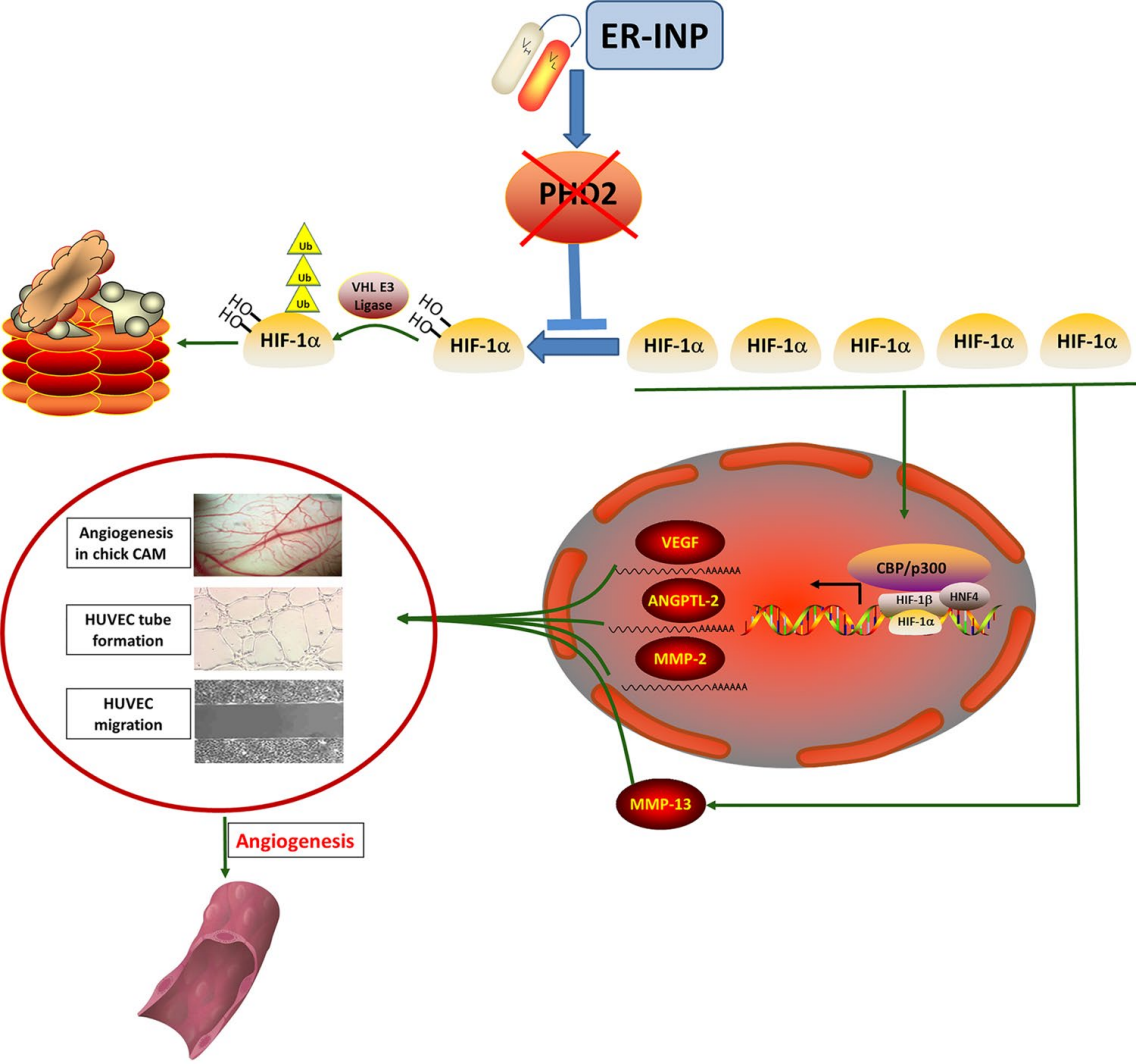
Molecular mechanism of ER-INP anti-PHD2’s promotion of angiogenesis via HIF-1α stabilization and transactivation under normoxia. ER-INP intrabody binds to PHD2 and blocks PHD2 hydroxylase activity to prevent the hydroxylation, ubiquitination, and degradation of HIF-1α. HIF-1α accumulation forms dimers by binding with HIF-1β and transactivates target genes at the consensus hypoxia-responsive elements 5′-(A/G) GGTG-3′. HIF-1α target genes control numerous key cellular processes, such as angiogenesis and metabolism. HIF-1α accumulation can promote MMP-13 expression, which can also promote angiogenesis.

In summary, this study has demonstrated that ER-INP binds to PHD2 and induces the functional knockdown of PHD2, thereby promoting accumulation and activation of HIF-1α and contributing to the proangiogenic effect. These results confirm the feasibility of a novel approach for therapeutic angiogenesis (Fig. 7).

## Materials and Methods

### Materials

Anti-β-actin antibody (A5441) was obtained from Sigma Chemical Co. (St. Louis, MO, USA). The antibodies against PHD2 (sc-271835) and VEGF (sc-152), normal mouse IgG antibody (sc-2025), and Protein A/G PLUS-Agarose (sc-2003), were purchased from Santa Cruz Biotechnology (Santa Cruz, CA). Anti-HIF-1α antibody (NB100-134) was purchased from Novus Biologicals (USA), anti-HIF-1α Pro402 antibody (07-1585) from Millipore (USA), anti-HIF-1α Pro564 antibody (D43B5) from Cell Signaling Technology (USA), rabbit anti-E-tag antibody (ab3397) from Abcam (UK), anti-β-tubulin antibody (K10322) from TransGen Biotech Co., LTD (Beijing, China), rabbit anti-PHD2 antibody (bs-3686R) from Bioss (Beijing, China), FITC-conjugated goat anti-rabbit IgG (HH012-1) from Beijing Dingguo Changsheng Biotechnology Co., Ltd. (Beijing, China), and mouse anti-E-tag antibody (EM50104) from HuaBio Inc. (Hangzhou, China). HEK293, HepG2, RAW264.7, and HUVEC cell lines were generously provided by Professor Xun Zhu (Jilin University, China).

### Construction of an anti-PHD2 intrabody

A human anti-PHD2 single-chain Fv antibody (INP) was screened from a human semi-synthetic scFv phage library (generously provided by Professor Lijun Zhou, Navy General Hospital, Beijing, China) by utilizing recombinant human PHD2 protein as a target. To efficiently block PHD2 activity in cells, anti-PHD2 intrabody (ER-INP) was prepared. In brief, using INP as a template, the PCR fragment of *ER-INP* was synthesised using primers harbouring the endoplasmic reticulum (ER) retention signal peptide SEKDEL and an E-tag sequence, and then the above PCR fragment was inserted into pcDNA3.1-FH (+) (constructed by Xiangzhi Liao, Jilin University). This completed the construction of the expression plasmid pER-INP harbouring an ER-retained scFv gene against human PHD2 fused with an additional E-tag.

### Cell culture and transfection

HEK293, HepG2, RAW264.7, and HUVEC cells were grown in Dulbecco’s modified Eagle’s medium (Gibco Life Technologies, MA, USA) supplemented with 10% foetal bovine serum. These cells were grown at 37 °C in 5% CO_2_. HEK293, HepG2, and RAW264.7 cells were grown in six-well plates and then transfected with plasmids (pER-INP or the control vector pcDNA3.1-FH) using different transfection reagents. HEK293 cells were transfected with calcium phosphate transfection reagent (DNA:transfection reagent ratio was 1:5) and RAW264.7 and HepG2 cells were transfected with jetPRIME® transfection reagent (Polyplus Transfection, FR) in accordance with the manufacturer’s instructions (DNA:transfection reagent ratio was 1:2). After transfection, the expression and subcellular distribution of ER-INP in HEK293, HepG2, and RAW264.7 cells was determined by RT-PCR, western blot, and IF analyses.

### Immunofluorescence staining analysis (IF)

HepG2 and RAW264.7 cells were cotransfected with the pDsRed2-ER plasmids (provided by Dr. Xuexun Fang, Jilin university) and control vector or ER-INP plasmids. At 48 hours post-transfection, the cells were digested and spread on the slides to culture for 4 hours. Cells on the slides were fixed with ethanol for 10 min at 4 °C, followed by permeabilisation with 0.05% Triton X-100 for 10 min and then blocked with PBS containing 3% BSA for 20 min. Cells were incubated with anti-E-tag polyclonal antibodies detecting ER-INP intrabodies at 4 °C overnight and then incubated with FITC-conjugated goat anti-rabbit IgG for 20 min at room temperature. DAPI, a fluorescent probe for DNA binding, was used to stain nuclei before observation under a microscope. The immunostained cells were examined with an Olympus IX73 fluorescence microscope and photographed using its auto-exposure mode.

### Western blot

Whole-cell lysates of HEK293 and RAW264.7 cells transfected with the control vector or pER-INP were extracted in a modified NP-40 lysis buffer with 1 mM dithiothreitol (DTT) and protease inhibitor cocktail tablets by freezing and thawing three times. The extracts were collected by centrifugation at 14,000 rpm and 4 °C for 10 min to remove cell debris and then were stored at −80 °C until use. The protein concentrations were determined by the Bradford assay. Equal amounts of protein were subjected to SDS-PAGE and the separated proteins were electrophoretically transferred onto amicroporous polyvinylidene difluoride (PVDF) membranes (Roche Applied Science). Nonspecific binding was blocked with PBS-T (0.05% Tween 20 in PBS) containing 5% non-fat milk for 1 hour at room temperature. The membranes were then incubated overnight at 4 °C with antibodies against PHD2, E-tag, VEGF, MMP-2, MMP-13, β-tubulin, and β-actin in PBS-T containing 5% non-fat milk at the dilutions specified by the manufacturers. Following three washes with PBS-T, the membranes were then incubated with horseradish peroxidase-conjugated secondary antibodies at a 1:5,000 dilution in PBS-T containing 5% non-fat milk for 1 hour at room temperature. The membranes were then washed three times with PBS-T and protein bands were visualised by EasySee Western Blot Kit (TransGen Biotech) and imaged with a Tanon 5200 automatic chemiluminescence image analysis system. Additionally, TBS-T (0.05% Tween 20 in TBS) was used during the western blot assay for HIF-1α, HIF-1α Pro402, and HIF-1α Pro564 according to the manufacturer’s instructions.

### Immunoprecipitation assay

For the CO-IP studies, whole-cell lysates of HEK293 cells transfected with the control vector or pER-INP were extracted by a modified Nonidet P-40 lysis buffer (150 mM NaCl, 1.0% NP-40, and 50 mM Tris, pH 8.0) containing protease inhibitor cocktail tablets. The supernatant was incubated with anti-E-tag antibody or normal mouse IgG antibody for 4 hours at 4 °C and then supplemented with Protein A/G PLUS-Agarose overnight at 4 °C. Proteins were separated by SDS-PAGE and analysed by western blot.

### Reverse-transcription PCR (RT-PCR) and quantitative real-time PCR (RT-qPCR) analysis

Total RNA from cells was extracted using TriPure Isolation Reagent (Roche). RNA purities and concentrations were assessed using a nanodrop spectrophotometer. RNA from the cells was reverse-transcribed to cDNA in accordance with the instructions provided with the reverse-transcription kit (GoScript™ Reverse Transcriptase; Promega, USA); this cDNA was subjected to RT-PCR, which was applied to determine the expression of ER-INP. In addition, PrimeScript RT Reagent Kit with gDNA Eraser was used to reverse-transcribe RNA from cells into cDNA and then amplify it using SYBR Premix Ex TaqII (TaKaRa, Dalian, China) and gene-specific primers for *β-actin*, *VEGF*, *ANGPTL-2*, *MMP-2*, and *MMP-13* (Table 1). All qPCR was performed using an Applied Biosystems 7500 Real-Time PCR system. The relative expression of the targeted gene was analysed by deducting its corresponding endogenous control threshold cycle (Ct) values according to the △△Ct method using the comparative Ct method normalised to β-actin expression. Data are presented as fold inductions of expression compared with those in the control conditions.

**Table 1:** Primer sequences of quantitative real-time polymerase chain reaction.

Genes	Sequences
β-actin	F: 5′-ACCAACTGGGACGACATGGA-3′
	R: 5′-GGTCTCAAACATGATCTGGGTCAT-3′
VEGF	F: 5′-GCACATAGAGAGAATGAGCTTCC-3′
	R: 5′-CTCCGCTCTGAACAAGGCT-3′
ANGPTL-2	F: 5′-AGCCTGAGAATACCAACCGC-3′
	R: 5′-CCCTTTGCTTATAGGTCTCCCAG-3′
MMP-2	F: 5′-CAAGGACCGGTTTATTT GGC-3′
	R: 5′-ATTCCCTGCGAAGAACACAGC-3′
MMP-13	F: 5′-CTTCTTCTTGTTGAGCTGGACTC-3′
(Mouse)	R: 5′-CTGTGGAGGTCACTGTAGACT-3′
MMP-13	F: 5′-GGTGGTGATGAAGATGATT-3′
(Human)	R: 5′-TCAGTCATGGAGCTTGCT-3′

### HUVEC migration and tube formation assay

Migration and tube formation of vascular endothelial cells in angiogenesis are two important events. The migration of HUVECs was examined by the wound healing assay. In order to detect the effect of ER-INP on migration of the HUVECs, we used 24-well plates with 6.5 mm diameter inserts (Transwell, Corning Inc., NY, USA) to co-culture HUVECs and HEK293 cells or RAW264.7 cells. HUVECs were harvested and plated on 24-well plates at a density of 2.5 × 10^5^/mL in 500 µL. When HUVECs formed a fully confluent monolayer on 24-well plates, the cells were starved overnight to be synchronized. The cells were then wounded by scratching with pipette tips and washed three times with D-Hanks buffer. Subsequently, HEK293 or RAW264.7 cells transfected with the control vector or pER-INP for 24 hours were digested and added to the upper chamber of the inserts (50,000 cells/well) and co-cultured with HUVECs for various lengths of time. Images were captured by an inverted microscope imaging system (Olympus DP74, Japan) after 0, 6, 12, and 18 hours of incubation. The width and closure rate of the wounds were measured.

To test whether ER-INP can promote HUVEC tube formation *in vitro*, a three-dimensional capillary tube formation assay was conducted. HEK293 and RAW264.7 cells transfected with the control vector or pER-INP for 24 hours were seeded into a 48-well plate coated with Matrigel Basement Membrane Matrix (BD Biosciences, USA) at a density of 10^3^ cells/well; simultaneously, HUVECs were seeded in the same 48-well plate at a density of 10^4^ cells /well. After incubation for 6, 12, and 24 hours, randomly chosen microscopic fields were photographed using an inverted microscope (Olympus DP74, Japan), and the length of closed networks of vessel-like tubes and the total branch lengths were analysed using ImageJ software.

### Assessment of ER-INP effect on angiogenesis in chick CAM

Fertilised Leghorn eggs were obtained from Changchun Institute of Biological Products and incubated in a humidified incubator at a temperature of 37.8 °C and relative humidity of 70%. Shells of 7-day-old embryonated eggs^58^ were cleaned with 75% ethanol and then a 2 cm^2^ window was opened above the air chamber. Fifty microliters of culture supernatant of cells transfected with the control vector or pER-INP for 48 hours was injected into a small hole made in the air chamber of the egg; then, the eggs were sealed with sterile adhesive tape. After treatment, the embryos were further incubated at 37.8 °C for two days. Next, each embryo was photographed using a Canon camera and six embryos were employed for each treatment group. The areas occupied by the blood vessels were quantified using Image Pro Plus Statistics Software, which automatically assesses and quantifies the blood vessel area^59^. The number of branches of the maximum blood vessel was quantified manually.

### Measurement of soluble pro-angiogenic mediators

The levels of VEGF, MMP-2, and MMP-13 in the cultured media from transfected HEK293 or RAW264.7 cells were measured using enzyme-linked immunosorbent assay (ELISA) kits according to the manufacturer’s instructions **(**Elabscience, Wuhan, China**)**.

### Statistical analysis

In this study, all values are presented as the mean ± SD and were analysed using SPSS 16.0 software. The statistically significant difference between two individual groups was determined with the two-tailed Student’s t-test. Differences with a *P* < 0.05 were considered significant.

## Supplementary information

Intrabody against prolyl hydroxylase 2 promotes angiogenesis by stabilizing hypoxia-inducible factor-1α

## Data Availability

No datasets were generated or analysed during the current study.

## References

[1] Semenza GL, Wang GL (1992). A nuclear factor induced by hypoxia via de novo protein synthesis binds to the human erythropoietin gene enhancer at a site required for transcriptional activation. Molecular and cellular biology.

[2] Koh MY (2016). A new HIF-1alpha/RANTES-driven pathway to hepatocellular carcinoma mediated by germline haploinsufficiency of SART1/HAF in mice. Hepatology.

[3] Xie C (2017). Activation of intestinal hypoxia-inducible factor 2alpha during obesity contributes to hepatic steatosis. Nat Med.

[4] Tak E (2017). Protective role of hypoxia-inducible factor-1alpha-dependent CD39 and CD73 in fulminant acute liver failure. Toxicol Appl Pharmacol.

[5] Nakayama T (2013). Role of macrophage-derived hypoxia-inducible factor (HIF)-1alpha as a mediator of vascular remodelling. Cardiovasc Res.

[6] You Q (2013). Role of hepatic resident and infiltrating macrophages in liver repair after acute injury. Biochem Pharmacol.

[7] Maxwell PH (1999). The tumour suppressor protein VHL targets hypoxia-inducible factors for oxygen-dependent proteolysis. Nature.

[8] Ohh M (2000). Ubiquitination of hypoxia-inducible factor requires direct binding to the beta-domain of the von Hippel-Lindau protein. Nature cell biology.

[9] Tian YM (2011). Differential sensitivity of hypoxia inducible factor hydroxylation sites to hypoxia and hydroxylase inhibitors. The Journal of biological chemistry.

[10] Hirsila M, Koivunen P, Gunzler V, Kivirikko KI, Myllyharju J (2003). Characterization of the human prolyl 4-hydroxylases that modify the hypoxia-inducible factor. The Journal of biological chemistry.

[11] Kwon HS (2011). Inhibition of a prolyl hydroxylase domain (PHD) by substrate analog peptides. Bioorg Med Chem Lett.

[12] Bruick RK (2003). Oxygen sensing in the hypoxic response pathway: regulation of the hypoxia-inducible transcription factor. Genes & development.

[13] Di Conza G (2017). The mTOR and PP2A Pathways Regulate PHD2 Phosphorylation to Fine-Tune HIF1alpha Levels and Colorectal Cancer Cell Survival under Hypoxia. Cell Rep.

[14] Berra E (2003). HIF prolyl-hydroxylase 2 is the key oxygen sensor setting low steady-state levels of HIF-1alpha in normoxia. The EMBO journal.

[15] Bubendorf L, Nocito A, Moch H, Sauter G (2001). Tissue microarray (TMA) technology: miniaturized pathology archives for high-throughput *in situ* studies. The Journal of pathology.

[16] Pu J (2019). Propofol Alleviates Apoptosis Induced by Chronic High Glucose Exposure via Regulation of HIF-1alpha in H9c2 Cells. Oxidative medicine and cellular longevity.

[17] Donahower B (2006). Vascular endothelial growth factor and hepatocyte regeneration in acetaminophen toxicity. American journal of physiology. Gastrointestinal and liver physiology.

[18] Kato T (2011). Vascular endothelial growth factor receptor-1 signaling promotes liver repair through restoration of liver microvasculature after acetaminophen hepatotoxicity. Toxicological sciences: an official journal of the Society of Toxicology.

[19] Zhang X (2018). Donor Treatment With a Hypoxia-Inducible Factor-1 Agonist Prevents Donation After Cardiac Death Liver Graft Injury in a Rat Isolated Perfusion Model. Artificial organs.

[20] Abe Y (2012). Liver epithelial cells proliferate under hypoxia and protect the liver from ischemic injury via expression of HIF-1 alpha target genes. Surgery.

[21] Okumura CY (2012). A new pharmacological agent (AKB-4924) stabilizes hypoxia inducible factor-1 (HIF-1) and increases skin innate defenses against bacterial infection. J Mol Med (Berl).

[22] Ivan M (2002). Biochemical purification and pharmacological inhibition of a mammalian prolyl hydroxylase acting on hypoxia-inducible factor. Proceedings of the National Academy of Sciences of the United States of America.

[23] Izuhara Y (2008). A novel class of advanced glycation inhibitors ameliorates renal and cardiovascular damage in experimental rat models. Nephrol Dial Transplant.

[24] Zaman K (1999). Protection from oxidative stress-induced apoptosis in cortical neuronal cultures by iron chelators is associated with enhanced DNA binding of hypoxia-inducible factor-1 and ATF-1/CREB and increased expression of glycolytic enzymes, p21(waf1/cip1), and erythropoietin. The Journal of neuroscience: the official journal of the Society for Neuroscience.

[25] Schreiber T, Salhofer L, Quinting T, Fandrey J (2019). Things get broken: the hypoxia-inducible factor prolyl hydroxylases in ischemic heart disease. Basic research in cardiology.

[26] Tanaka T, Eckardt KU (2018). HIF Activation Against CVD in CKD: Novel Treatment Opportunities. Seminars in nephrology.

[27] Sugahara M, Tanaka T, Nangaku M (2017). Prolyl hydroxylase domain inhibitors as a novel therapeutic approach against anemia in chronic kidney disease. Kidney international.

[28] Zhou C, Przedborski S (2009). Intrabody and Parkinson’s disease. Biochimica et biophysica acta.

[29] Scott AM, Wolchok JD, Old LJ (2012). Antibody therapy of cancer. Nat Rev Cancer.

[30] Marschall AL, Dubel S, Boldicke T (2015). Specific *in vivo* knockdown of protein function by intrabodies. mAbs.

[31] Reimer E (2013). Molecular cloning and characterization of a novel anti-TLR9 intrabody. Cellular & molecular biology letters.

[32] Meli G, Visintin M, Cannistraci I, Cattaneo A (2009). Direct *in vivo* intracellular selection of conformation-sensitive antibody domains targeting Alzheimer’s amyloid-beta oligomers. Journal of molecular biology.

[33] Van Impe K (2013). A nanobody targeting the F-actin capping protein CapG restrains breast cancer metastasis. Breast cancer research: BCR.

[34] Sakuma C, Sato M, Takenouchi T, Chiba J, Kitani H (2012). Single-chain variable fragment intrabody impairs LPS-induced inflammatory responses by interfering with the interaction between the WASP N-terminal domain and Btk in macrophages. Biochem Biophys Res Commun.

[35] Koivunen P, Serpi R, Dimova EY (2016). Hypoxia-inducible factor prolyl 4-hydroxylase inhibition in cardiometabolic diseases. Pharmacological research.

[36] Metzen E (2003). Intracellular localisation of human HIF-1alpha hydroxylases: implications for oxygen sensing. Journal of Cell Science.

[37] Lee DH, Goldberg AL (1998). Proteasome inhibitors: valuable new tools for cell biologists. Trends in cell biology.

[38] Tennant DA (2009). Reactivating HIF prolyl hydroxylases under hypoxia results in metabolic catastrophe and cell death. Oncogene.

[39] Shweiki D, Itin A, Soffer D, Keshet E (1992). Vascular endothelial growth factor induced by hypoxia may mediate hypoxia-initiated angiogenesis. Nature.

[40] Zhou J (2016). Potential Role of Hyperglycemia in Fetoplacental Endothelial Dysfunction in Gestational Diabetes Mellitus. Cellular physiology and biochemistry: international journal of experimental cellular physiology, biochemistry, and pharmacology.

[41] Zhang Y, Shao Z, Zhai Z, Shen C, Powell-Coffman JA (2009). The HIF-1 hypoxia-inducible factor modulates lifespan in C. elegans. PLoS One.

[42] Geis T (2015). HIF-2alpha-dependent PAI-1 induction contributes to angiogenesis in hepatocellular carcinoma. Exp Cell Res.

[43] Feng N (2016). HIF-1alpha and HIF-2alpha induced angiogenesis in gastrointestinal vascular malformation and reversed by thalidomide. Scientific reports.

[44] Cheng X (2016). Dexamethasone Exposure Accelerates Endochondral Ossification of Chick Embryos Via Angiogenesis. Toxicological sciences: an official journal of the Society of Toxicology.

[45] Siamblis D (1996). A novel radiological approach for the experimental study of angiogenesis: angiography of the chick embryo and its chorioallantoic membrane. European journal of radiology.

[46] Ha MK (2011). Emodin inhibits proinflammatory responses and inactivates histone deacetylase 1 in hypoxic rheumatoid synoviocytes. Biological & pharmaceutical bulletin.

[47] West JB (2017). Physiological Effects of Chronic Hypoxia. N Engl J Med.

[48] Rajagopalan S, Rane A, Chinta SJ, Andersen JK (2016). Regulation of ATP13A2 via PHD2-HIF1alpha Signaling Is Critical for Cellular Iron Homeostasis: Implications for Parkinson’s Disease. The Journal of neuroscience: the official journal of the Society for Neuroscience.

[49] Kaelin WG, Ratcliffe PJ (2008). Oxygen sensing by metazoans: the central role of the HIF hydroxylase pathway. Mol Cell.

[50] Loinard C (2009). Inhibition of prolyl hydroxylase domain proteins promotes therapeutic revascularization. Circulation.

[51] Suhara T (2015). Inhibition of the oxygen sensor PHD2 in the liver improves survival in lactic acidosis by activating the Cori cycle. Proceedings of the National Academy of Sciences of the United States of America.

[52] Marschall AL, Dubel S (2016). Antibodies inside of a cell can change its outside: Can intrabodies provide a new therapeutic paradigm?. Computational and structural biotechnology journal.

[53] Cardinale A, Biocca S (2008). The potential of intracellular antibodies for therapeutic targeting of protein-misfolding diseases. Trends in molecular medicine.

[54] Munro S, Pelham HR (1987). A C-terminal signal prevents secretion of luminal ER proteins. Cell.

[55] Lederle W (2010). MMP13 as a stromal mediator in controlling persistent angiogenesis in skin carcinoma. Carcinogenesis.

[56] Ye Y (2014). Inhibition of epidermal growth factor receptor signaling prohibits metastasis of gastric cancer via downregulation of MMP7 and MMP13. Tumour biology: the journal of the International Society for Oncodevelopmental Biology and Medicine.

[57] Pufe T (2004). Mechanical overload induces VEGF in cartilage discs via hypoxia-inducible factor. The American journal of pathology.

[58] Hamburger V, Hamilton HL (1992). A series of normal stages in the development of the chick embryo. 1951. Developmental dynamics: an official publication of the American Association of Anatomists.

[59] Larger E (2003). [Hyperglycemia and angiogenesis]. Med Sci (Paris).

