# Supplementary material for: Intrabody against prolyl hydroxylase 2 promotes angiogenesis by stabilizing hypoxia-inducible factor-1α
Source: Sci Rep. 2019 Aug 14;9:11861. doi: 10.1038/s41598-019-47891-1 (PMC6694103; doi:10.1038/s41598-019-47891-1)
Supplement: Supplementary file 1 — Intrabody against prolyl hydroxylase 2 promotes angiogenesis by stabilizing hypoxia-inducible factor-1α [file 41598_2019_47891_MOESM1_ESM.pdf]

Supplementary information

Intrabody against prolyl hydroxylase 2 promotes angiogenesis by stabilizing hypoxia-inducible factor-1α

Liangzhong Zhao[1,2], Ziyu Liu[1], Fang Yang[1], Ying Zhang[1,3], Ying Xue[1], Haipeng Miao[1], Xiangzhi Liao[1], Hongli Huang[1], Guiying Li[1]*

1 Key Laboratory for Molecular Enzymology and Engineering of the Ministry of Education, School of Life Sciences, Jilin University, Changchun 130012, China;

2 Department of Immunoassay Technology, Jilin Medical University, Jilin 132013, China;

3 Department of Pediatrics, The First Hospital of Jilin University, Changchun, Jilin 130021, P.R. China;

* **Corresponding author:** Guiying Li, Key Laboratory for Molecular Enzymology and Engineering of the Ministry of Education, School of Life Sciences, Jilin University, Changchun 130012, China. Tel.: +86-431-85155222 E-mail: ligy@jlu.edu.cn.

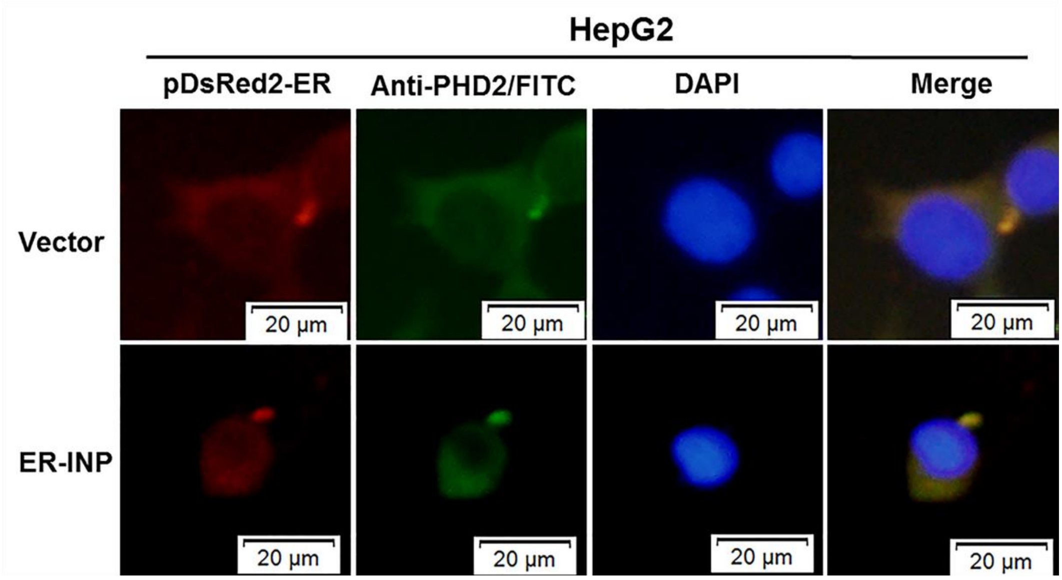

Supplement Figure 1. Intracellular localization of PHD2 by Immunofluorescence assay in HepG2 cells. pDsRed2-ER and PHD2 are co-localized in the endoplasmic reticulum.

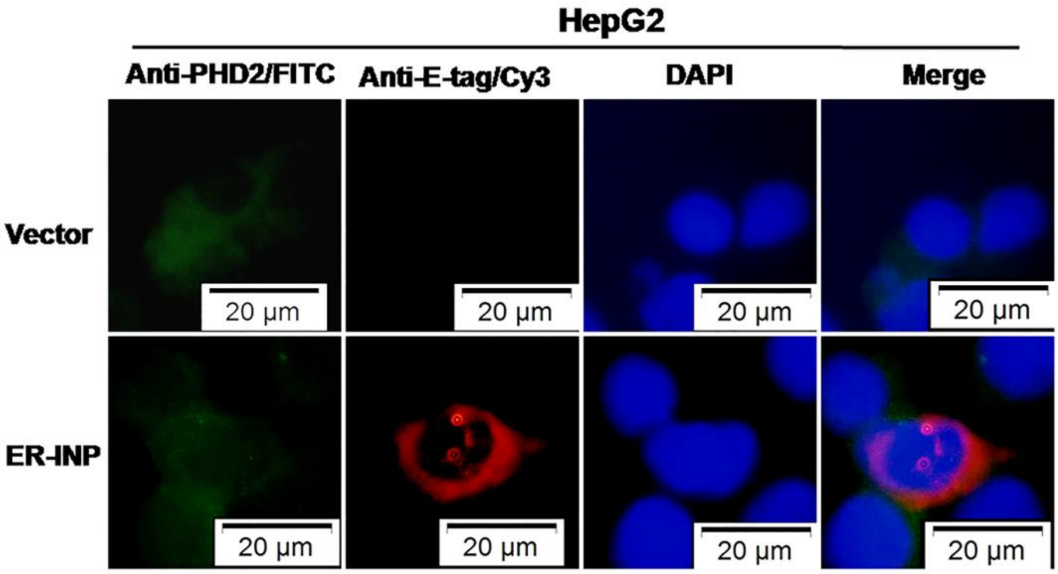

Supplement Figure 2. Intracellular localization of PHD2 and ER-INP by Immunofluorescence assay. PHD2 and ER-INP are co-localized in the endoplasmic reticulum.

The data used to support the findings of this article is as follows.

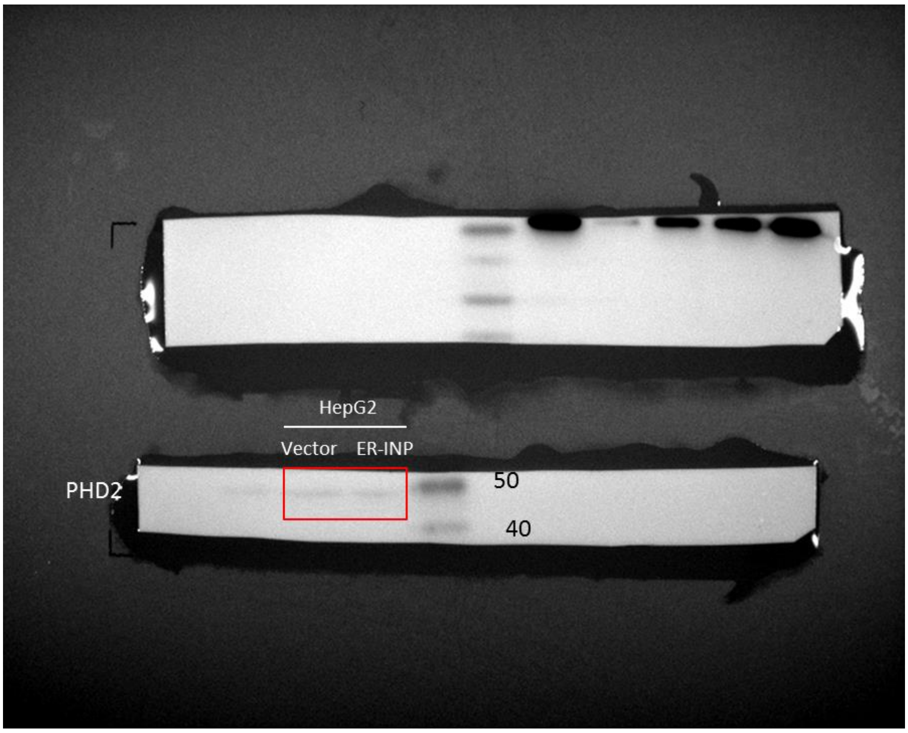

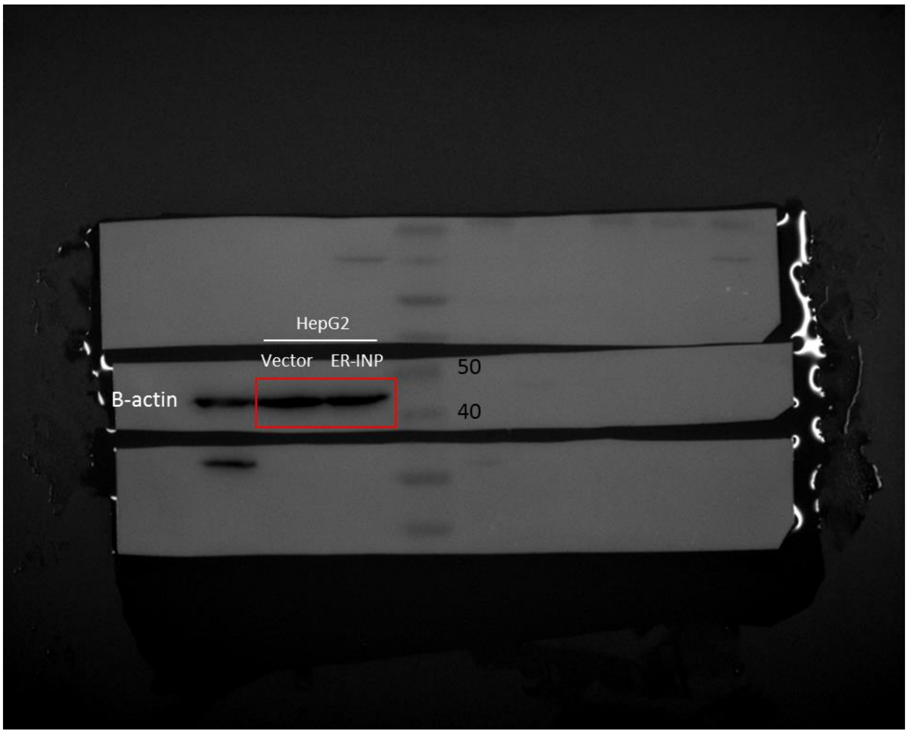

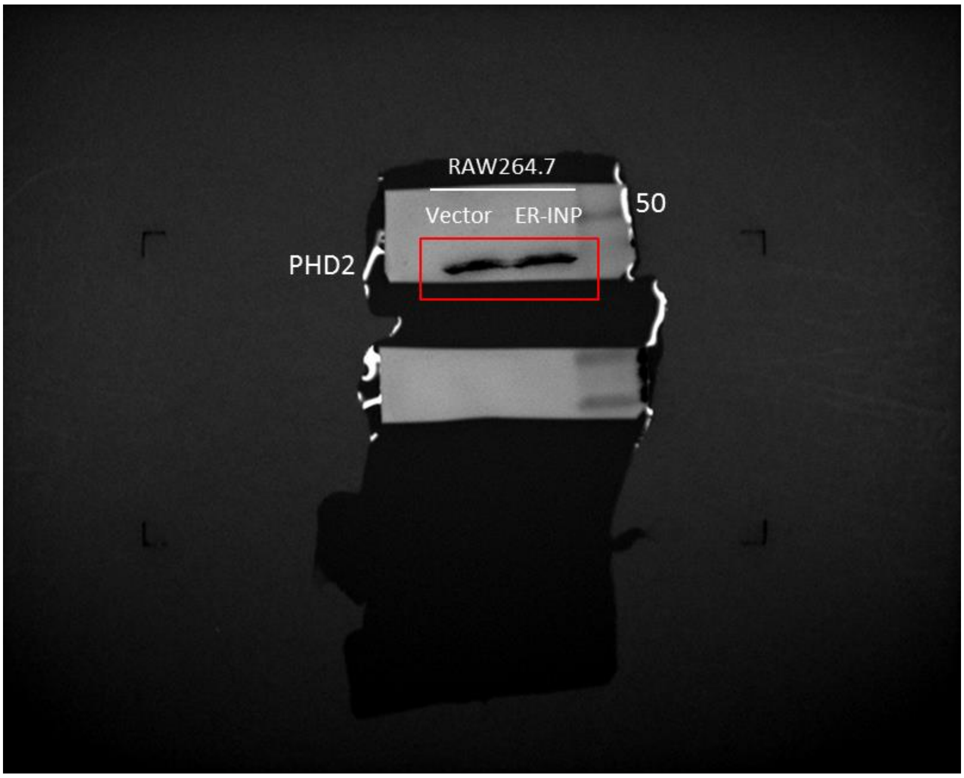
RAW264.7
Vector
ER-INP
50
PHD2

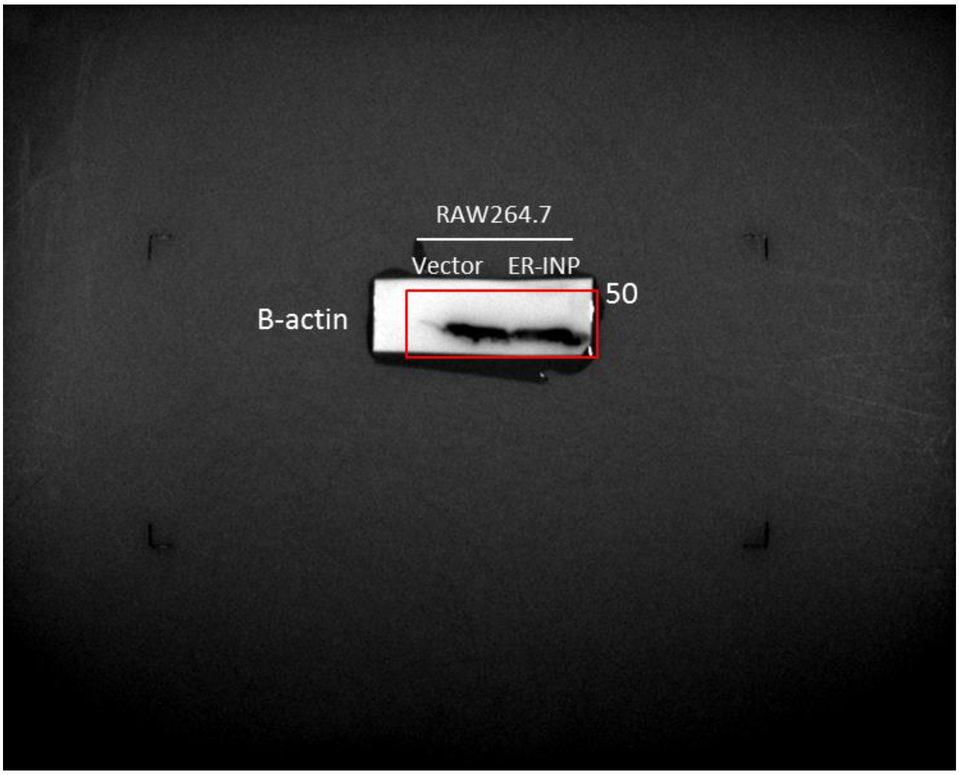
RAW264.7
Vector
ER-INP
50
B-actin

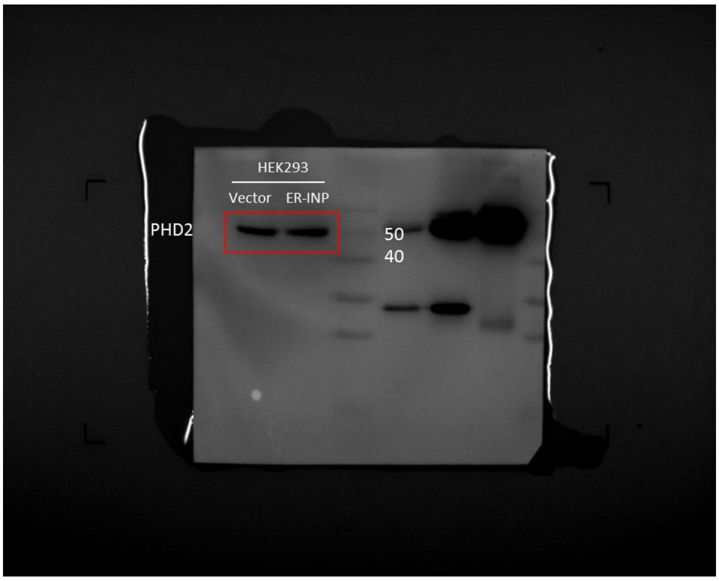
HEK293
Vector ER-INP
PHD2
50
40

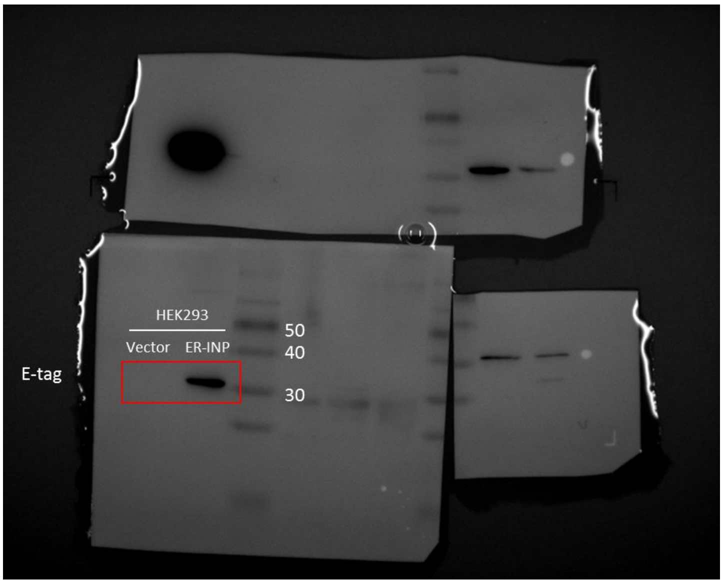
HEK293
Vector ER-INP
E-tag
50
40
30

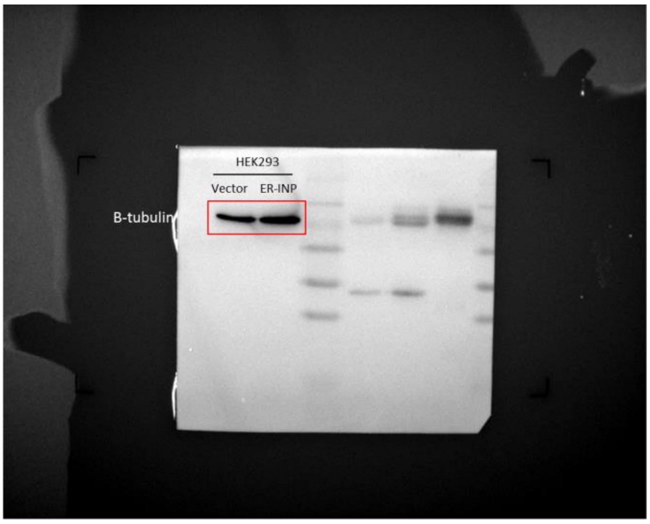
HEK293
Vector ER-INP
B-tubulin

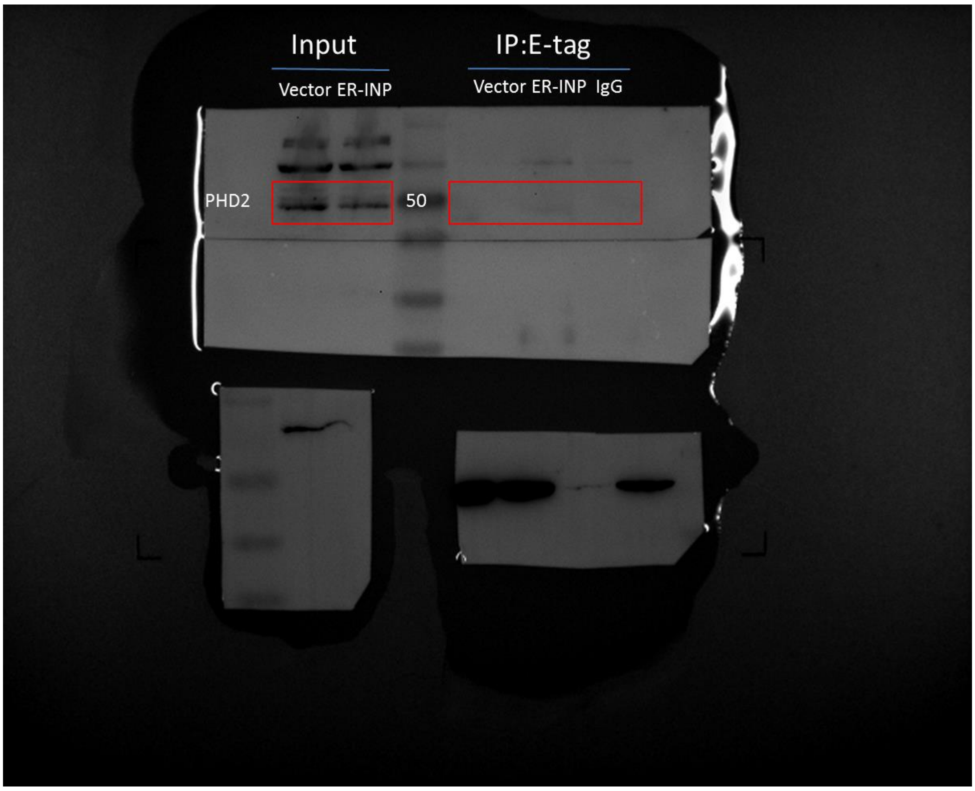
Input
IP:E-tag
Vector ER-INP
Vector ER-INP IgG
PHD2
50

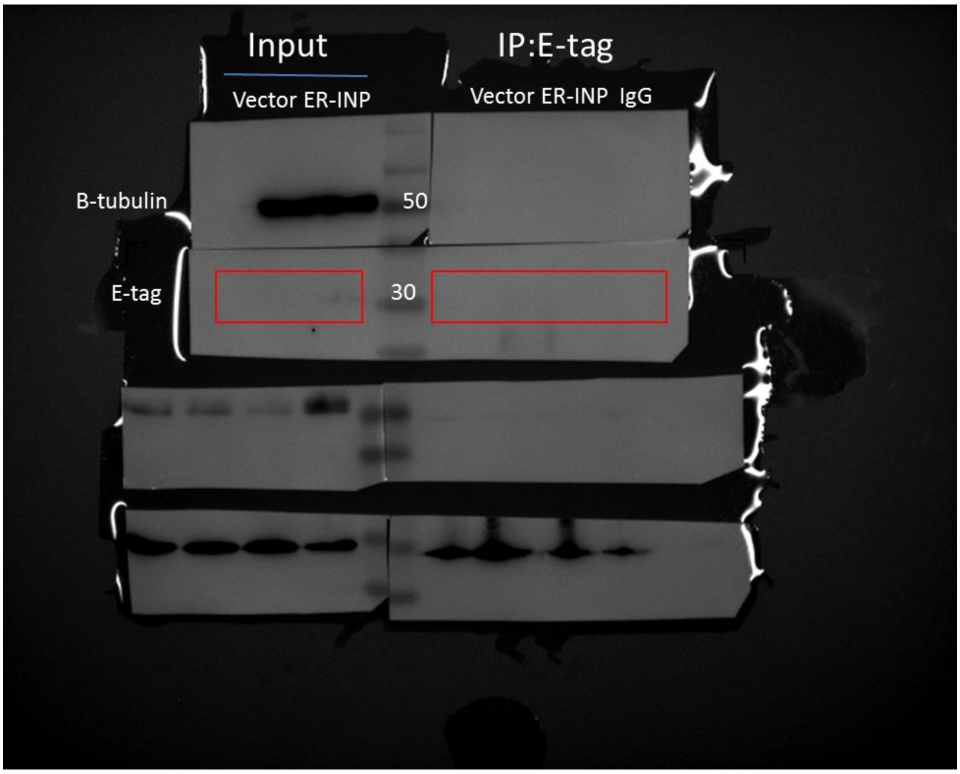
Input
IP:E-tag
Vector ER-INP
Vector ER-INP IgG
B-tubulin
50
E-tag
30

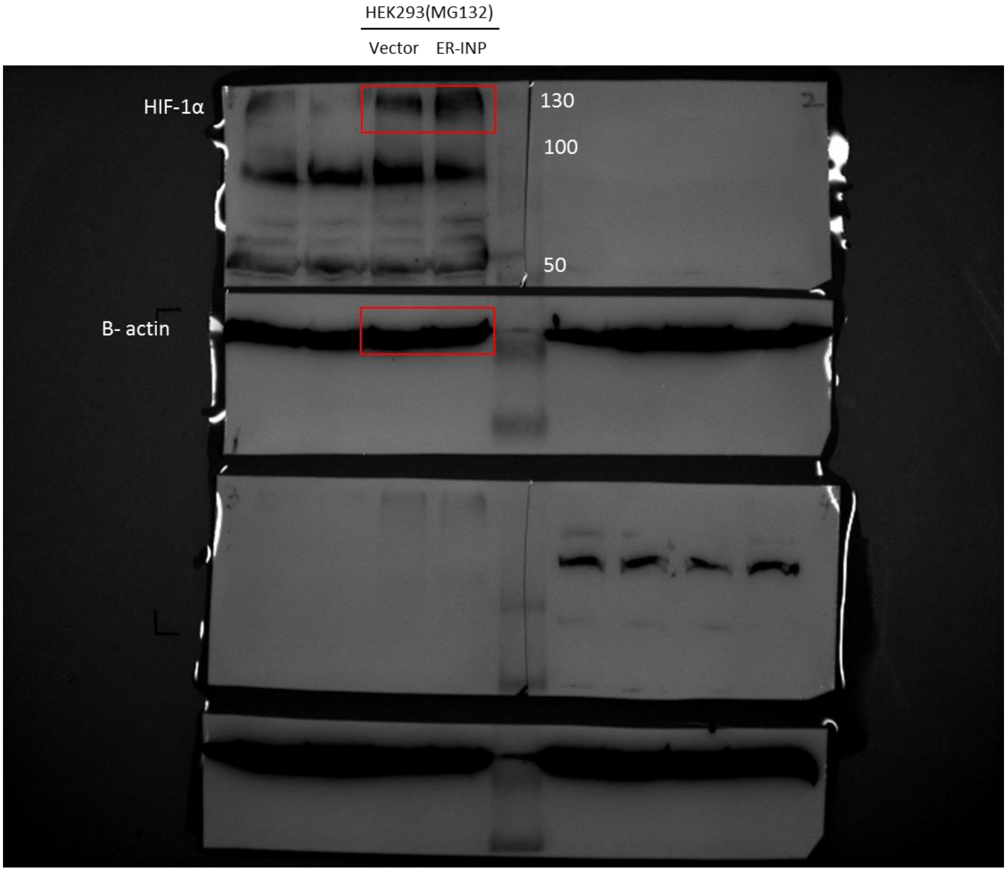
HEK293(MG132)
Vector ER-INP
HIF-1α
130
100
50
B- actin

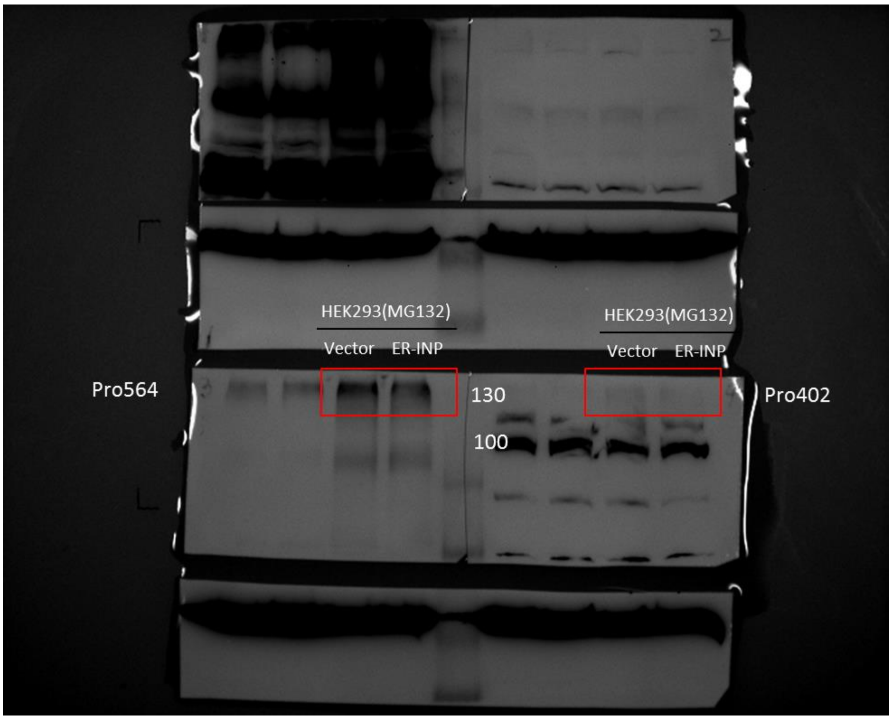
HEK293(MG132)
Vector ER-INP
HEK293(MG132)
Vector ER-INP
Pro564
130
100
Pro402

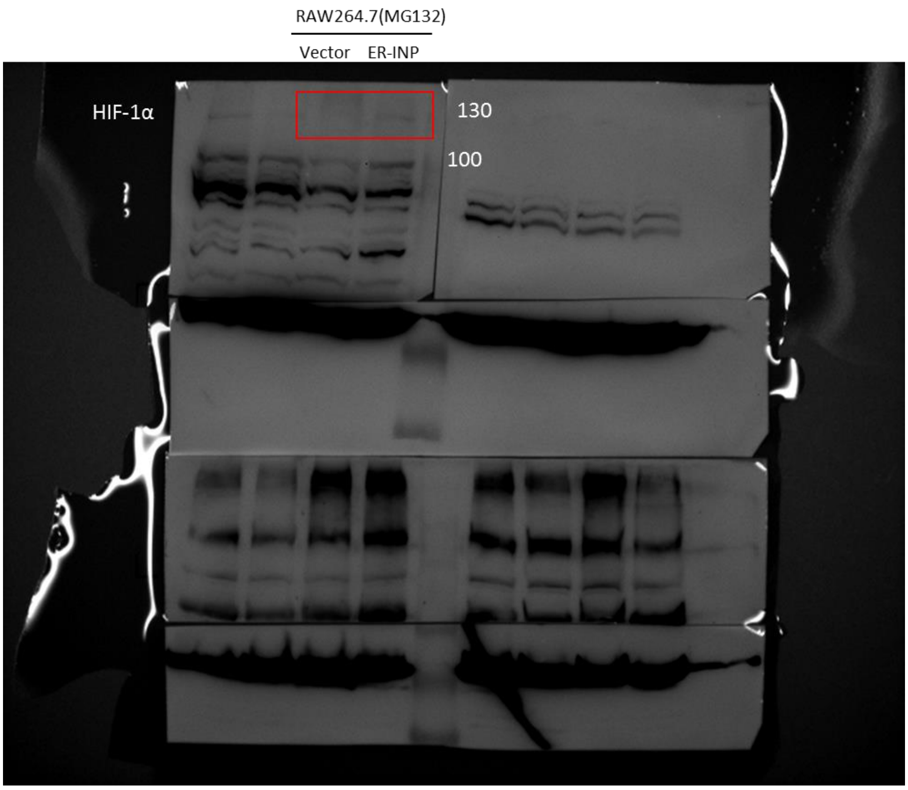

RAW264.7(MG132)
Vector ER-INP
HIF-1α
130
100

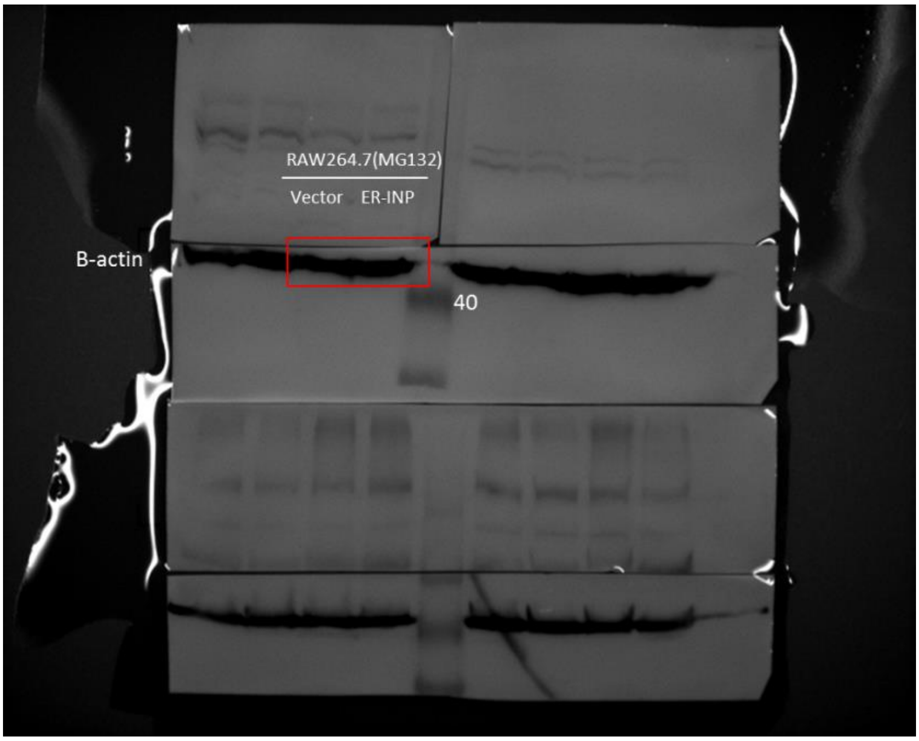

RAW264.7(MG132)
Vector ER-INP
B-actin
40

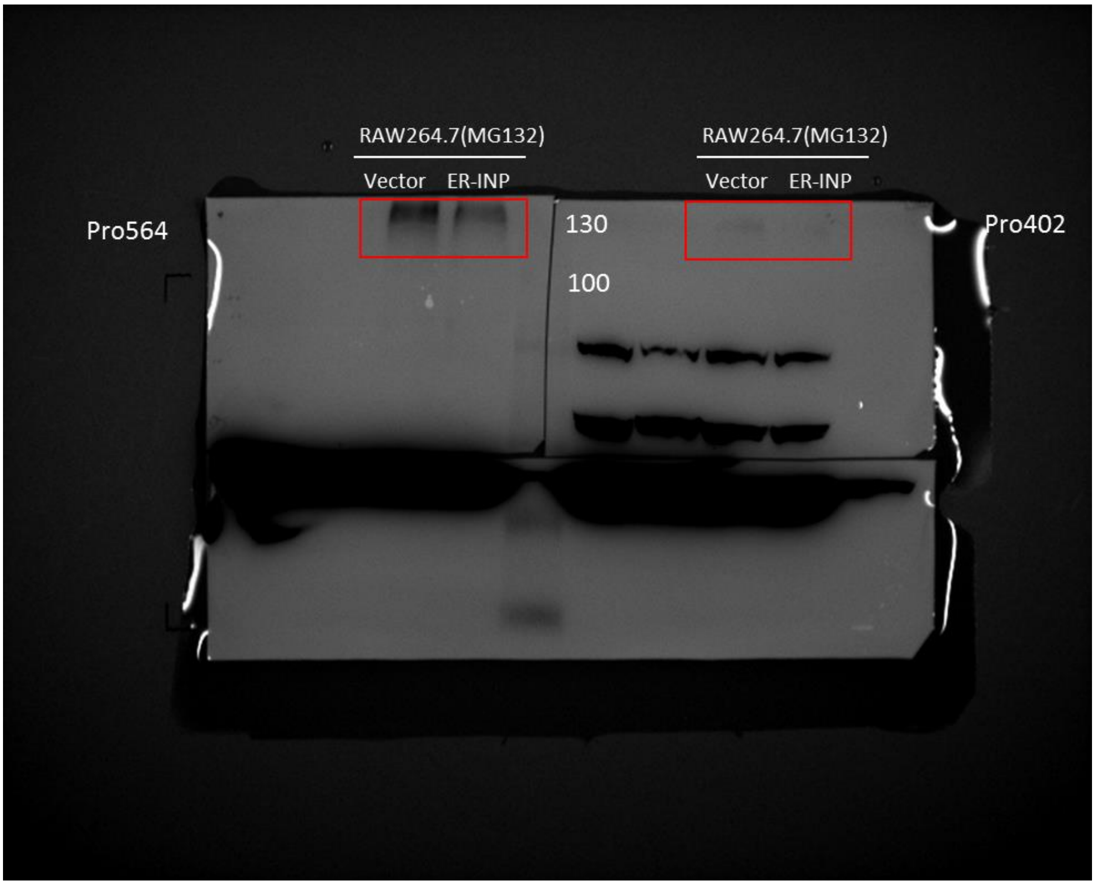
RAW264.7(MG132)
Vector ER-INP
RAW264.7(MG132)
Vector ER-INP
Pro564
130
100
Pro402

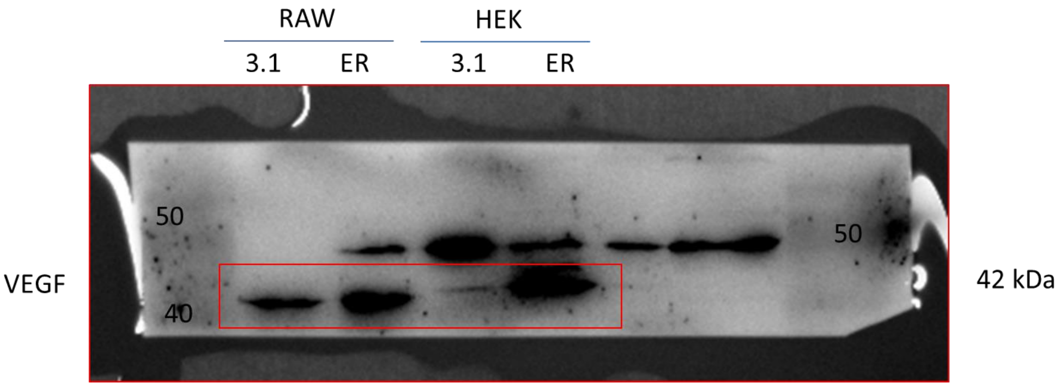
RAW
HEK
3.1 ER 3.1 ER
VEGF
50
40
50
42 kDa

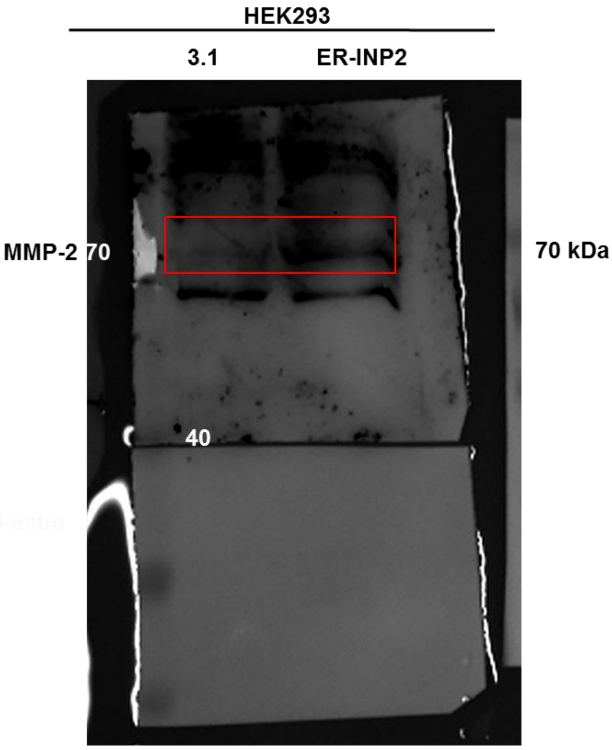
HEK293
3.1
ER-INP2
MMP-2 70
70 kDa
40

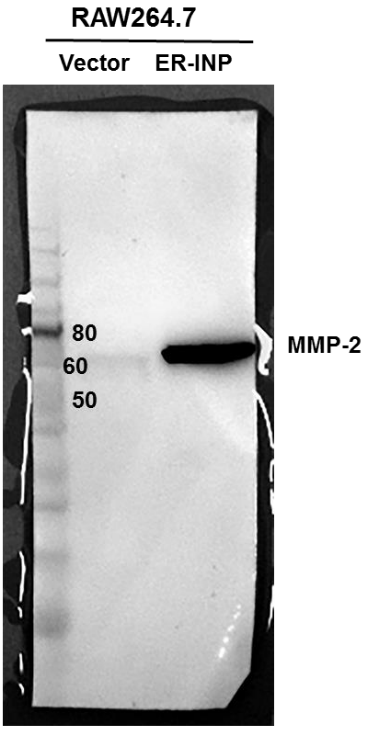
RAW264.7
Vector
ER-INP
80
60
50
MMP-2

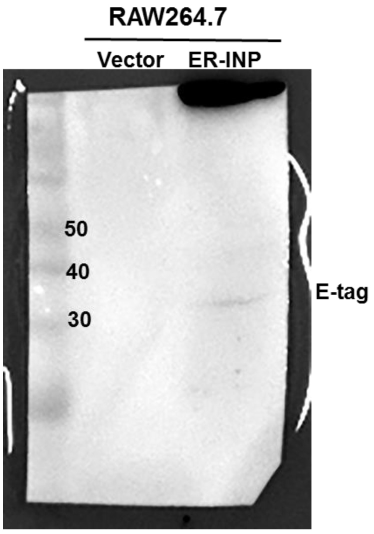
RAW264.7
Vector
ER-INP
50
40
30
E-tag

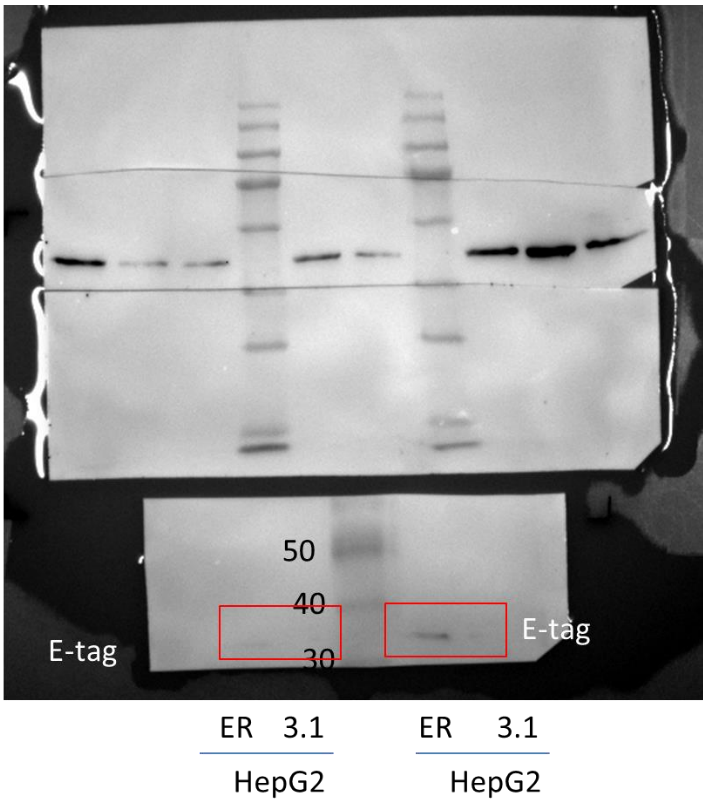
50
40
30
E-tag
E-tag
ER
3.1
ER
3.1
HepG2
HepG2
